# An Explainable Machine Learning-Based QSAR Framework for Predicting Thrombin Inhibitory Activity

**DOI:** 10.3390/ph19091411

**Published:** 2026-09-07

**Authors:** Ali Onur Kaya, Mert Can Emre

**Affiliations:** 1 Radiotherapy Department, Health Services Vocational School, Akdeniz University, 07070 Antalya, Türkiye; 2 Motor Vehicles Department, Sorgun Vocational School, Yozgat Bozok University, 66000 Yozgat, Türkiye; m.can.emre@yobu.edu.tr

**Keywords:** thrombin inhibitors, quantitative structure–activity relationship (QSAR), explainable artificial intelligence, ensemble learning, drug discovery

## Abstract

**Background/Objectives:** Public thrombin bioactivity records contain heterogeneous endpoints, replicate measurements, related chemical series, and potentially reactive compounds that may bias quantitative structure–activity relationship models. In this study, we developed an explainable, assay-aware, and leakage-safe machine learning framework for predicting thrombin-inhibitory activity. **Methods:** Exact *K_i_* and IC_50_ records for human thrombin (CHEMBL204) were standardized, converted to pActivity, and aggregated using predefined criteria. The final dataset comprised 5189 unique compounds represented by development-filtered Mordred descriptors and Morgan fingerprint. The models were optimized using development-only out-of-fold validation and evaluated using random and scaffold-disjoint held-out tests. **Results:** In the random-split analysis, ConsensusAll achieved an out-of-fold R^2^ of 0.7588 and a held-out test R^2^ of 0.7480, with an RMSE of 0.7360, MAE of 0.5307, and concordance correlation coefficient of 0.8540. In the scaffold-disjoint locked test, ConsensusTop5 achieved R^2^ = 0.5831, RMSE = 0.9484, and MAE = 0.7290, respectively. The applicability domain covered 92.68% of the locked test compounds and yielded R^2^ = 0.6041. One hundred Y-randomization runs produced a mean R^2^ of −0.1233 (empirical *p* = 0.0099). **Conclusions:** The framework provides useful predictions within the represented chemical space and measurable generalization for unseen scaffolds. This supports compound prioritization, although prospective biochemical validation remains necessary.

## 1. Introduction

Hemostasis is a tightly regulated physiological process that preserves vascular integrity through a dynamic balance between procoagulants and anticoagulant mechanisms. Following vascular injury, platelet activation and sequential activation of coagulation factors culminate in thrombin generation and the formation of a stable fibrin clot. Disruption of this balance may result in either excessive bleeding or pathological thrombosis, emphasizing the importance of precise regulation of the coagulation cascade. Thrombin (coagulation factor IIa), a trypsin-like serine protease generated from the zymogen prothrombin, occupies a central position in this process because it contributes to clot initiation, amplification, and stabilization [1,2,3,4].

The biological functions of thrombin extend beyond the proteolytic conversion of fibrinogen to fibrin. Thrombin amplifies coagulation by activating factors V, VIII, XI, and XIII and promotes platelet activation primarily through protease-activated receptors PAR-1 and PAR-4. Conversely, after binding to thrombomodulin, thrombin contributes to anticoagulant regulation by activating the protein C pathway. Thrombin signaling is also involved in inflammation, endothelial function, vascular homeostasis, tissue remodeling, and wound healing. Consequently, excessive thrombin generation or dysregulated thrombin activity is implicated in thromboembolic and cardiovascular disorders, including deep-vein thrombosis, pulmonary embolism, ischemic stroke, and myocardial infarction [1,2].

The therapeutic relevance of thrombin has led to the development of direct thrombin inhibitors, including dabigatran, argatroban, and bivalirudin [5]. Unlike indirect anticoagulants, these agents inhibit thrombin without requiring antithrombin as a cofactor and have expanded the available options for the prevention and treatment of thromboembolic diseases. Nevertheless, their clinical use remains associated with limitations such as bleeding risk, pharmacokinetic variability, renal or hepatic dose-adjustment requirements, drug-specific administration constraints, and contraindications in particular patient populations [6,7,8,9]. Therefore, the discovery and optimization of thrombin inhibitors with improved potency, selectivity, pharmacokinetic properties, and safety profiles remain relevant objectives in medicinal chemistry and anticoagulant drug development [5,6,7,8,9].

Computer-aided drug discovery provides complementary strategies for identifying and prioritizing biologically active compounds before extensive experimental evaluation. Structure-based methods, including molecular docking and molecular dynamics simulations, use three-dimensional information about biological targets to investigate protein–ligand interactions. Ligand-based approaches infer biological activity from the structural and physicochemical properties of compounds with known experimental responses. Quantitative structure–activity relationship (QSAR) modeling is one of the most established ligand-based approaches and relates numerical representations of molecular structures to experimentally measured biological activities. When developed and validated appropriately, QSAR models can support virtual screening, compound prioritization, and exploration of structure–activity relationships within a defined chemical domain [10,11,12,13,14,15].

The availability of public bioactivity repositories has considerably expanded the chemical and biological scope of contemporary QSAR investigations. ChEMBL integrates chemical structures, target annotations, assay information, and experimentally reported bioactivity measurements extracted from the medicinal chemistry literature. These data facilitate the construction of larger and more chemically diverse datasets than the small congeneric series traditionally used in QSAR studies. However, publicly aggregated bioactivity records are inherently heterogeneous and may include duplicate structures, inconsistent molecular representations, missing information, multiple endpoint types, repeated measurements, and measurements generated under different experimental conditions. Therefore, systematic molecular standardization, endpoint definition, replicate handling, descriptor pre-processing, and transparent data curation are essential for developing reproducible models from such repositories [16,17,18,19].

The simultaneous presence of the inhibition constant (*K*i) and half-maximal inhibitory concentration (IC_50_) measurements presents an additional challenge in thrombin bioactivity modeling. Although both endpoints describe inhibitory potency, they are not experimentally or mechanistically equivalent to each other. *K*i is an inhibition constant associated with inhibitor–enzyme binding, whereas IC_50_ may vary according to substrate concentration, assay configuration, incubation time, and inhibition mechanism [20,21]. Consequently, the uncontrolled pooling of *K*i and IC_50_ measurements may introduce additional response heterogeneity. Nevertheless, these endpoints can be integrated cautiously when their identities are retained, replicate measurements are evaluated for concordance, highly discordant observations are excluded, and endpoint-specific sensitivity analyses are performed. Potentially reactive structural motifs should also be considered because some compounds may act through covalent or time-dependent inhibition. Structural alerts alone do not prove such mechanisms; however, their prevalence and influence on model performance should be examined.

Machine learning algorithms have extended QSAR modeling by enabling the capture of nonlinear relationships and interactions among molecular features without imposing a predefined functional form. Tree-based ensemble methods, including random forests, gradient-boosting algorithms, and extremely randomized trees, are particularly suitable for high-dimensional descriptor matrices and chemically heterogeneous ligand datasets [22,23]. Molecular descriptors and circular fingerprints may also provide complementary representations of chemical structures: descriptors summarize global constitutional, physicochemical, and topological properties, whereas fingerprints encode local atom environments and structural motifs [24]. Therefore, the combined evaluation of these representations can broaden the molecular information available to predictive algorithms [24,25,26,27].

Nevertheless, predictive performance obtained from a single data partition is insufficient to establish model reliability. Conventional random splitting may distribute closely related compounds or members of the same chemical series between the training and test sets, thereby producing optimistic estimates of the predictive performance. Therefore, held-out testing should be complemented by resampling-based validation and a more demanding structure-aware evaluation. Scaffold-disjoint partitioning, in which compounds sharing the same Bemis–Murcko scaffold are assigned to the same subset, provides a more stringent assessment of model generalization to molecular frameworks that have not been previously observed [25]. In addition, descriptor filtering, hyperparameter optimization, algorithm selection, and ensemble construction should be performed exclusively using the development data. A locked test set can provide an unbiased final assessment only when its responses are not used during the model development or candidate selection. Response randomization and applicability domain analysis provide complementary evidence by evaluating potential chance correlations and identifying the chemical space in which the predictions are structurally supported [26,27].

The increasing use of complex machine learning models has intensified the need for interpretable predictions. Although ensemble algorithms may achieve strong predictive performance, their internal decision structures do not directly reveal how individual molecular characteristics influence a specific prediction. Explainable artificial intelligence methods can help address this limitation. Among these methods, SHapley Additive exPlanations (SHAP) provides feature-level contributions for individual predictions and aggregated measures of global feature importance. In QSAR applications, SHAP can reveal the molecular information used by a fitted model while allowing the examination of nonlinear effects and feature interactions. For fingerprint-based models, influential fingerprint bits can also be mapped to representative atom environments, providing a more direct connection between the model attribution and molecular structure. However, such interpretations should be regarded as model-based associations rather than direct evidence of molecular mechanisms or causality [28,29,30,31].

Computational modeling of thrombin inhibitors has previously been investigated using statistical QSAR, molecular descriptors, machine-learning algorithms, and structure-based analyses. Earlier investigations frequently focused on relatively small congeneric compound series and local structure–activity relationships, whereas more recent studies have explored larger datasets, ensemble learning, and explainable modeling strategies. In particular, Masand et al. applied a genetic algorithm–XGBoost approach and SHAP-based interpretation to a structurally diverse dataset of thrombin inhibitors [32]. Their study demonstrated the potential of explainable machine learning for identifying molecular features associated with thrombin-inhibitory activity. The present study complements this earlier work by incorporating assay-aware activity harmonization, explicit replication and endpoint auditing, descriptor- and fingerprint-based molecular representations, development-only model selection, scaffold-disjoint locked testing, reactive-substructure sensitivity analysis, and comprehensive Supplementary Information. Therefore, the contribution of the present investigation lies not in the first application of machine learning or SHAP to thrombin inhibitors but in the integration of these methodological components within a unified and transparently validated framework [32,33].

In this study, an interpretable machine learning-based QSAR framework was developed to predict thrombin-inhibitory activity using systematically curated bioactivity records retrieved from ChEMBL. The experimental *K*i and IC_50_ measurements were converted to their corresponding negative logarithmic values and modeled as a combined inhibitory activity endpoint, referred to as pActivity. Replicate measurements and compounds with both endpoint types were processed using predefined concordance criteria, and their original endpoint identities were retained for subsequent sensitivity analyses. Following assay-aware activity harmonization and molecular standardization, the final regression dataset comprised 5189 unique compounds represented by two-dimensional Mordred descriptors and Morgan fingerprints.

Multiple regression algorithms and consensus models were optimized and selected using out-of-fold predictions generated exclusively from the development data set. Predictive performance was evaluated using two complementary validation strategies. First, a conventional random held-out partition was used to assess the performance within the represented chemical space and facilitate comparison with commonly reported QSAR results. Second, a more stringent scaffold-disjoint protocol was applied, in which complete Bemis–Murcko scaffold groups were separated between the development and locked test sets. The scaffold model was selected and frozen before the locked test responses were accessed. Model robustness and practical utility were further assessed using 100-run Y-randomization, similarity-based applicability domain analysis, endpoint-specific and reactive substructure sensitivity analyses, and SHAP-based structural interpretation. Therefore, this study aimed to establish a transparent, reproducible, and chemically informed ligand-based framework for prioritizing candidate thrombin inhibitors while distinguishing predictions within a known chemical space from generalization to previously unseen molecular scaffolds.

## 2. Results

### 2.1. Assay-Aware Curation and Dataset Characteristics

The initial ChEMBL export of human thrombin (CHEMBL204) contained 9327 bioactivity records [16]. A sequential assay-aware curation procedure was applied to retain experimentally interpretable and structurally valid data [17,18,19]. Restricting the records to an exact activity relation (“=”) reduced the dataset to 6849 entries, of which 6777 were reported in nanomolar units. The removal of records carrying ChEMBL data validity warnings yielded 6435 measurements. Molecular standardization and RDKit sanitization produced 6434 structurally valid records, and the removal of exact duplicate entries resulted in 6414 eligible activity measurements.

Among these records, 3988 were *K*i measurements representing 3445 unique standardized structures, and 2426 were IC_50_ measurements representing 2025 unique structures. Measurements were grouped according to the standardized molecular structure and original endpoint type before structure-level aggregation. A total of 4843 structure–endpoint groups contained single measurements. Among the groups containing replicate measurements, 451 were classified as highly consistent, with a maximum activity span of ≤0.5 logarithmic units, and 80 were classified as acceptable, with a span between 0.5 and 1.0 logarithmic units. Ninety-six structure–endpoint groups exhibited a span greater than 1.0 logarithmic units and were excluded as conflicting measurements. The median pActivity was used to represent each retained replicate. The principal dataset characteristics are summarized in Table 1, while the complete record-level curation flow, replicate, and endpoint audits are provided in Tables S1 and S2 and illustrated in Figures S1–S3.

Following endpoint-level aggregation, 3259 compounds were represented exclusively by K_i_ measurements, 1843 exclusively by IC_50_ measurements, and 136 structures had measurements available for both endpoints before cross-endpoint concordance filtering. For the 136 structures represented by both K_i_ and IC_50_ measurements, the absolute difference between the two structure-level median values was evaluated [20,21]. Forty-nine discordant cross-endpoint pairs exceeding the predefined threshold of 1.0 logarithmic unit were excluded, leaving 87 concordant dual-endpoint structures. The final pooled regression dataset therefore comprised 5189 unique thrombin inhibitors, including 3259 K_i_-only, 1843 IC_50_-only, and 87 concordant dual-endpoint compounds, with pActivity values ranging from 3.37 to 11.00. The endpoint composition of the final dataset and the agreement between paired structure-level K_i_ and IC_50_ measurements are presented in Figures S1 and S2, respectively.

Potentially reactive substructures were identified using predefined structural alerts. At least one alert was detected in 520 compounds, corresponding to 10.02% of the final dataset, whereas 4669 compounds contained none of the investigated motifs [34]. The most frequent alerts were boronic acids or boronic esters (*n* = 165), activated alkenes or Michael acceptor motifs (*n* = 154), α-ketoamides (*n* = 95), nitriles (*n* = 83), acrylamides (n = 80), aldehydes (*n* = 48), vinyl sulfones (*n* = 17), and sulfonyl fluorides (*n* = 1). Because more than one alert can occur within the same molecule, the sum of the individual alert counts exceeds the number of alerted compounds. These alerts were retained as sensitivity analysis labels and were not interpreted as direct evidence of covalent or time-dependent inhibition. The principal characteristics of the final curated dataset are presented in Table 1.

### 2.2. Molecular Representation and Leakage-Safe Feature Processing

Two complementary molecular representations were generated for the curated compounds in this study. Mordred initially calculated 1613 two-dimensional molecular descriptors [35]. Feature preprocessing was performed using only the development compounds. The application of the predefined missing-value threshold retained 1450 descriptors with no more than 20% missing observations. The removal of descriptors with zero or negligible variance reduced the descriptor matrix to 1312 variables. Pairwise redundancy filtering was then performed using a predefined absolute-correlation threshold of 0.95, leaving 615 nonredundant Mordred descriptors. Missing values in these retained descriptors were replaced using the medians calculated exclusively from the corresponding development data.

Morgan fingerprints were calculated with a radius of 2, a length of 2048 bits, and chirality information [24]. Nine fingerprint bits were constant across the development compounds and were removed, leaving 2039 informative Morgan features remaining. The descriptor lists, fingerprint-bit columns, descriptor-imputation values, and feature order were subsequently frozen and applied unchanged to the held-out subsets of the dataset. Accordingly, neither the random held-out responses nor the scaffold-disjoint locked-test responses contributed to the feature filtering, imputation, or molecular-representation processing. The principal feature-processing results are summarized in Table 2, while the complete development-only feature-filtering audit is provided in Table S3 and Figure S4 (Supporting Information).

### 2.3. Random Held-Out Model Development and Predictive Performance

A conventional random 80:20 partition was first employed to evaluate the predictive performance within the chemical space represented by the complete curated dataset and to facilitate comparison with the original model. The random development subset contained 4151 compounds, whereas 1038 compounds were reserved as held-out test sets. Model selection was conducted exclusively through five-fold out-of-fold predictions generated within the development subset. The randomly held-out responses were not used for descriptor pre-processing, hyperparameter optimization, algorithm ranking, or consensus-weight estimation.

Substantial differences were observed between the evaluated regression algorithms and molecular representations. Among the individual models, ExtraTreesMorgan [36] achieved the highest development out-of-fold performance, with R^2^ = 0.7448, RMSE = 0.7440, MAE = 0.5435, CCC = 0.8527, and Pearson’s *r* = 0.8634. XGBoostCombined [37] and LightGBMCombined [38] produced development out-of-fold R^2^ values of 0.7170 and 0.7080, respectively. The descriptor-based SVRMordred [39] model yielded R^2^ = 0.6931, whereas NuSVRMorgan produced R^2^ = 0.6406.

**Figure 1:**
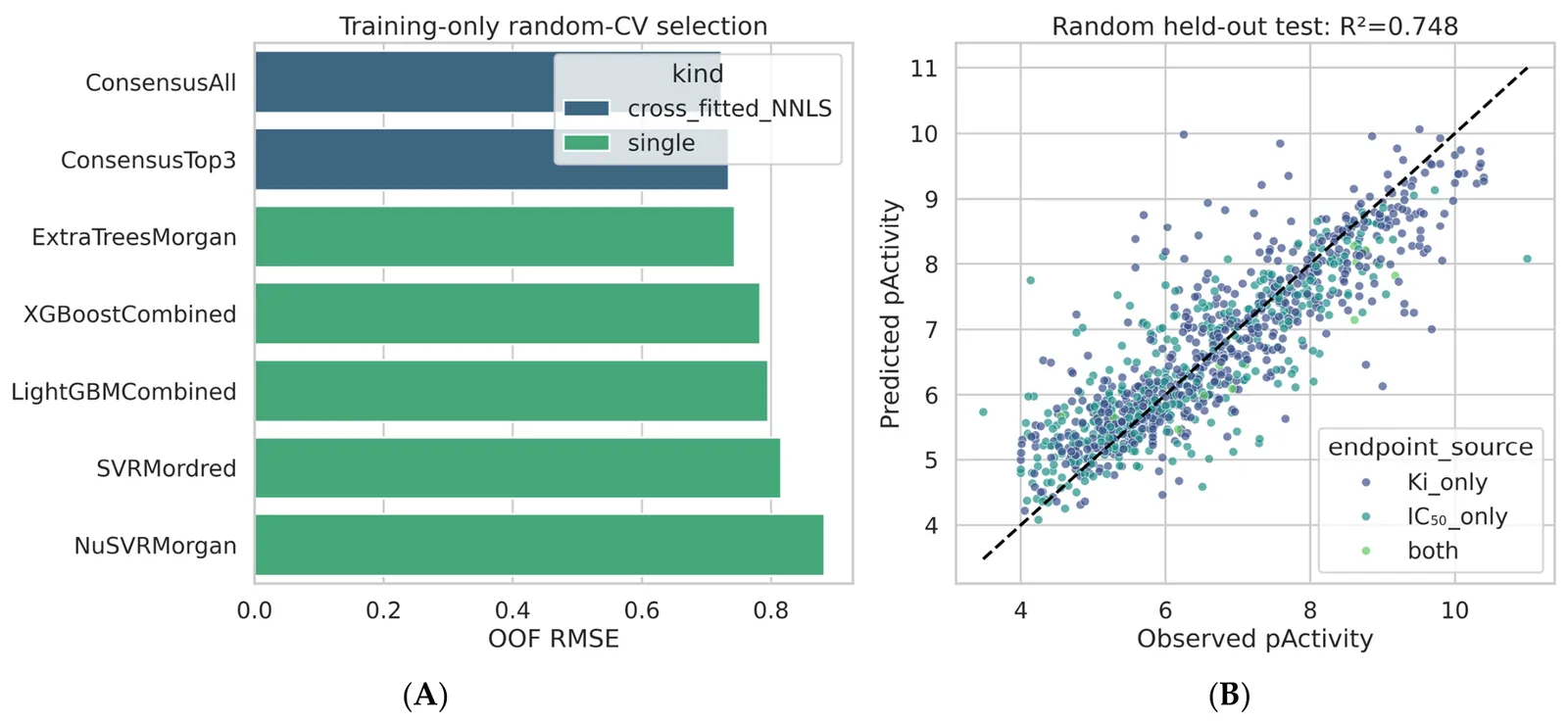
Random held-out confirmation of the development-selected ConsensusAll model. (**A**) Development of out-of-fold ranking of evaluated candidates. (**B**) Experimental versus predicted pActivity values for the 1038 randomly held-out compounds. The diagonal line represents y = x.

Cross-fitted non-negative consensus modeling improved the performance beyond that of the individual candidate algorithms [40]. ConsensusAll ranked first according to the development of the out-of-fold RMSE and achieved R^2^ = 0.7588, RMSE = 0.7234, MAE = 0.5334, CCC = 0.8571, and Pearson’s *r* = 0.8724. ConsensusTop3 ranked second, with R^2^ = 0.7510 and RMSE = 0.7349. The complete development of the out-of-fold ranking is shown in Table 3 and Figure 1A.

After candidate selection was completed, the frozen ConsensusAll model was evaluated against the 1038-compound random held-out test set. The model achieved R^2^ = 0.7480, with a bootstrap 95% confidence interval of 0.7107–0.7811 [41]. The corresponding RMSE was 0.7360 (95% CI: 0.6874–0.7848), MAE was 0.5307 (95% CI: 0.5007–0.5622), CCC was 0.8540, and Pearson’s *r* was 0.8653. The relationship between the experimental and predicted pActivity values is shown in Figure 1B, and the complete development of out-of-fold and held-out metrics is summarized in Table 4. The close correspondence between the development out-of-fold R^2^ = 0.7588 and random held-out R^2^ = 0.7480 indicated a stable predictive performance within the randomly partitioned chemical space.

### 2.4. Scaffold-Disjoint Dataset Partitioning

A second, substantially more stringent validation protocol was established to evaluate the model transfer to previously unseen molecular frameworks. Complete Bemis–Murcko scaffold groups [25] were assigned to either the development or locked test subset using exact mixed-integer linear programming. The resulting development set contained 4151 compounds representing 1180 scaffolds, whereas the locked test set contained 1038 compounds representing 295 scaffolds in the training set. No Bemis–Murcko scaffolds were shared between the two subsets.

The activity distributions were closely balanced among the scaffold partitions. The mean pActivity was 6.5613 in the development set and 6.5598 in the locked test set, with corresponding standard deviations of 1.4723 and 1.4697, respectively. The development set contained 2607 *K*i-only compounds, 1474 IC_50_-only compounds, and 70 compounds with both endpoints. The locked test set contained 652 *K*i-only compounds, 369 IC_50_-only compounds, and 17 compounds with concordant information from both the endpoints. Potential reactive alerts were present in 416 development compounds and 104 locked test compounds, corresponding to almost identical proportions in both subsets. The principal partition characteristics are summarized in Table 5, while the complete partition-balance audit is provided in Table S4 and illustrated in Figures S5 and S6 of the Supporting Information.

The median maximum Morgan Tanimoto similarity of the locked test compounds to the development set was 0.7045. Thus, although the locked test compounds did not share complete Bemis–Murcko scaffolds with the development compounds, portions of their local structural environments were represented to varying degrees. Five additional scaffold-grouped folds were defined within the development set for hyperparameter optimization and candidate selection. Each scaffold was confined to one-fold, preventing compounds with the same core framework from appearing simultaneously in the fitting and validation portions of an internal cross-validation iteration. The locked test-to-development similarity distribution and scaffold-allocation audit are presented in Figure S5.

### 2.5. Scaffold-Grouped Model Selection and Locked-Test Performance

All candidate models in the scaffold-disjoint analysis were optimized and compared using only the five predefined scaffold-grouped folds in the development set. The locked test file was not accessed during the descriptor preprocessing, hyperparameter optimization, algorithm ranking, or ensemble construction.

Under scaffold-grouped out-of-fold validation, the individual model performance was lower than that obtained with random cross-validation. ExtraTreesMorgan remained the strongest individual candidate and achieved R^2^ = 0.5714, RMSE = 0.9637, MAE = 0.7330, CCC = 0.7245, and Pearson’s *r* = 0.7602. XGBoostCombined produced R^2^ = 0.5447, whereas LightGBMCombined and NuSVRMorgan achieved R^2^ values of 0.5369 and 0.5204, respectively.

The cross-fitted ConsensusTop5 model ranked first in the scaffold-grouped development analysis, with R^2^ = 0.5983, RMSE = 0.9331, MAE = 0.7230, CCC = 0.7268, and Pearson’s *r* = 0.7845. ConsensusAll achieved a closely comparable R^2^ of 0.5975 and RMSE of 0.9340, whereas ConsensusTop3 produced R^2^ = 0.5834 and RMSE = 0.9502, respectively. ConsensusTop5 was selected based on its development of out-of-fold RMSE and was frozen before the locked-test responses were accessed. The principal scaffold-grouped development results are presented in Table 6, whereas the complete model ranking and hyperparameter-optimization records are provided in Tables S5 and S6 and Figure S7.

**Figure 2:**
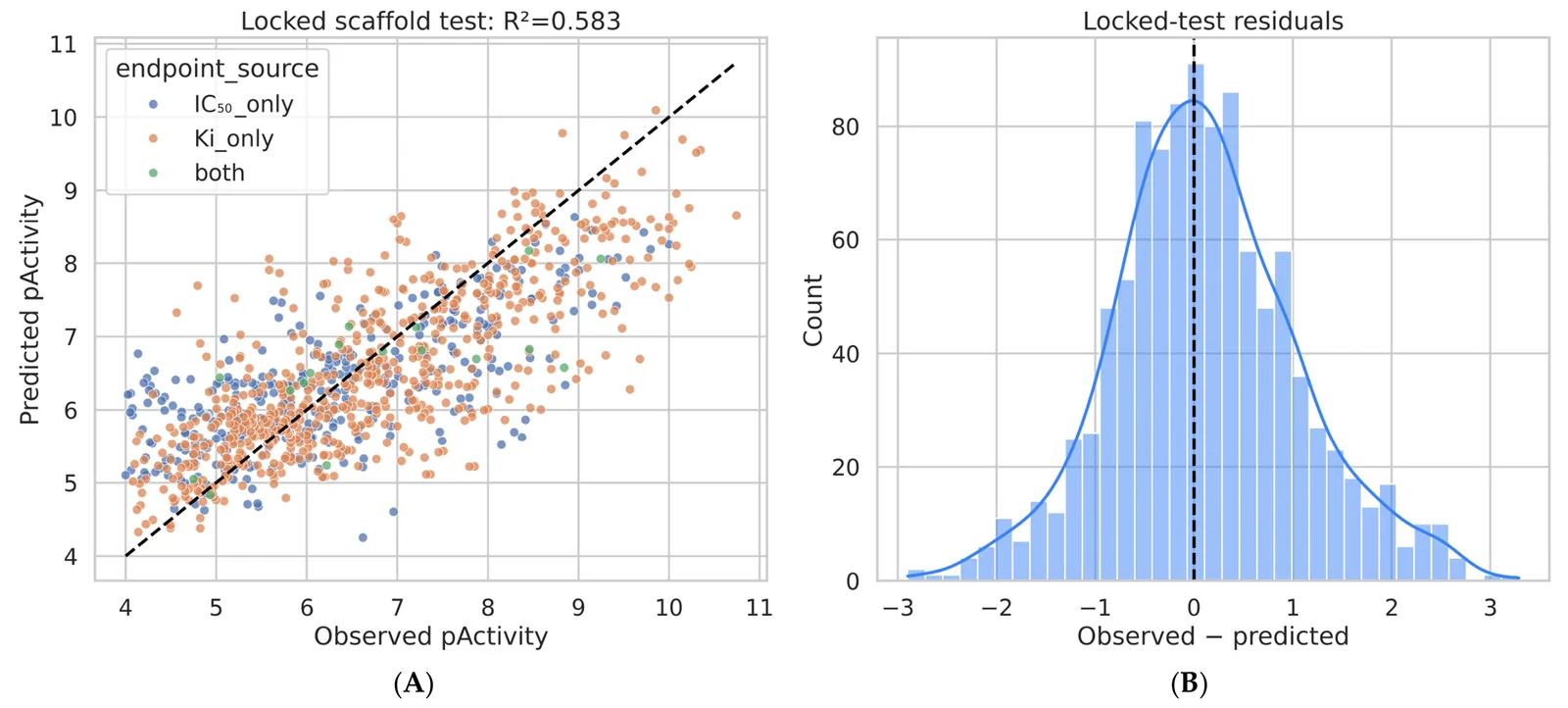
One-time evaluation of the frozen ConsensusTop5 model on the scaffold-disjoint locked test set. The 295 test scaffolds were absent from the development set. (**A**) Experimental versus predicted pActivity values; the black dashed reference line represents y = x, and the colors indicate the original endpoint sources. (**B**) Distribution of observed-minus-predicted residuals; the black dashed vertical reference line indicates zero residual, and the blue curve represents the kernel density estimate.

In the one-time evaluation of the 1038-compound scaffold-disjoint locked test set, the frozen ConsensusTop5 model achieved R^2^ = 0.5831, with a bootstrap 95% confidence interval of 0.5424–0.6198. The corresponding RMSE was 0.9484 (95% CI: 0.9028–0.9922), MAE was 0.7290 (95% CI: 0.6941–0.7671), CCC was 0.7257, and Pearson’s *r* was 0.7693. These values were close to the development scaffold-grouped out-of-fold results, for which the R^2^ and RMSE were 0.5983 and 0.9331, respectively. The relationship between the experimental and predicted pActivity values in the locked scaffold test is shown in Figure 2, and the development and locked-test metrics are summarized in Table 7. No post-test modifications of the selected model, molecular representations, feature sets, hyperparameters, or consensus composition were performed.

### 2.6. Comparison of Random and Scaffold-Disjoint Validation

The random and scaffold-disjoint analyses produced clearly different estimates of the predictive performance. The random held-out model achieved R^2^ = 0.7480, whereas the frozen scaffold model achieved R^2^ = 0.5831 in the locked scaffold test. The absolute difference between the two estimates was 0.1649. The RMSE increased from 0.7360 in the random held-out test to 0.9484 in the scaffold-disjoint test, while the MAE increased from 0.5307 to 0.7290.

A corresponding difference was observed during the development-only validation. Random out-of-fold validation of ConsensusAll produced R^2^ = 0.7588, whereas scaffold-grouped out-of-fold validation of ConsensusTop5 produced R^2^ = 0.5983. The close correspondence between each development out-of-fold result and its respective held-out result indicated that the performance difference originated primarily from the structural difficulty of the partitioning strategy rather than from instability after the final model fitting.

The random held-out result exceeded the R^2^ = 0.7246 obtained for the ExtraTrees model in the original analysis. However, the scaffold-disjoint result provided a separate and more demanding estimate of the transfer to compounds with previously unseen core frameworks. Accordingly, the random and scaffold results were retained as complementary evaluations rather than being combined into a single performance statistic. The comparative performances of the random and scaffold-disjoint protocols are summarized in Table 4 and Table 7 and visually compared in Figure 1 and Figure 2.

### 2.7. Endpoint-Specific and Reactive-Substructure Sensitivity Analyses

Scaffold-disjoint locked-test predictions were stratified according to the original experimental endpoint. For the 652 *K*i-only compounds, the frozen consensus model achieved an R^2^ of 0.6053. For the 369 IC_50_-only compounds, R^2^ was 0.5097. The subgroup containing 17 compounds represented by concordant information from both endpoint types yielded R^2^ = 0.5060; however, this result was interpreted cautiously because of the limited size of the subgroup.

Endpoint-specific refitting of the dominant ExtraTreesMorgan component provided complementary assessment. The model trained using the available *K*i subset achieved R^2^ = 0.6197 in the corresponding scaffold-disjoint evaluation, whereas the IC_50_-specific model achieved R^2^ = 0.4711. These findings showed that the predictive signal was retained for both endpoint types, although the performance was consistently higher for *K*i than for IC_50_ measurements.

**Figure 3:**
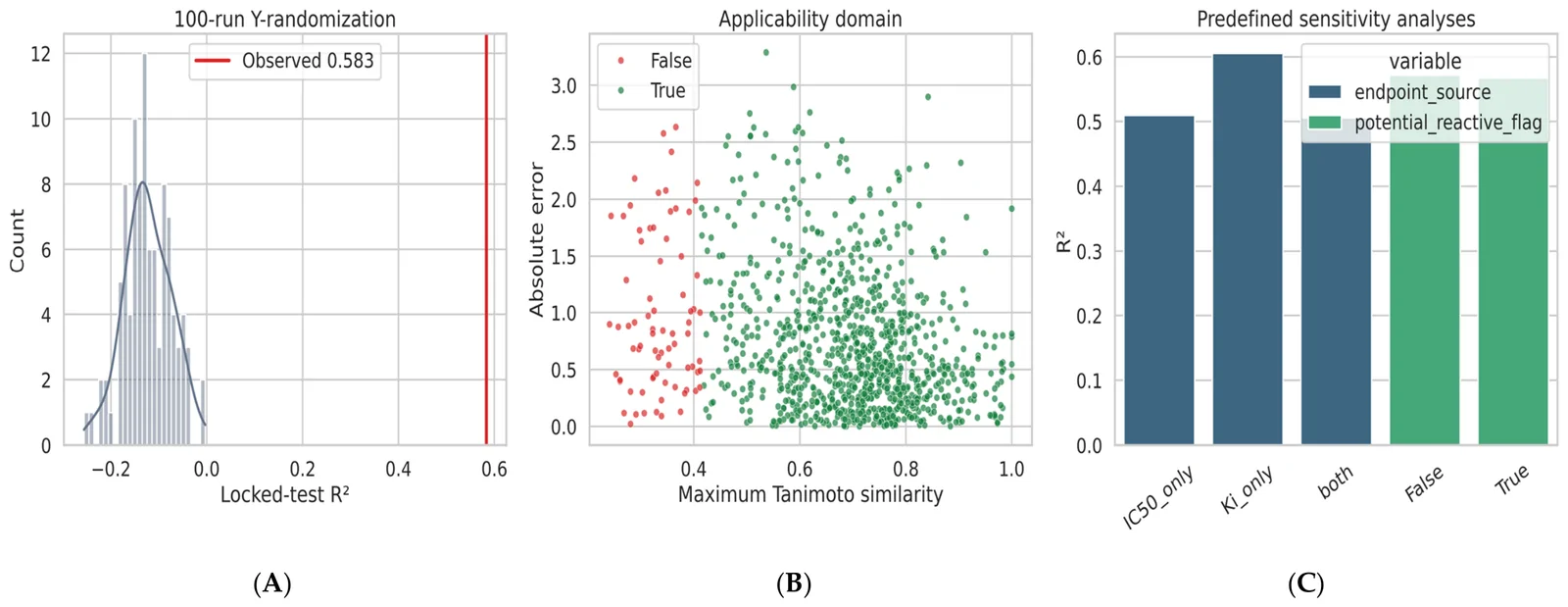
Robustness and applicability-domain assessment. (**A**) Distribution of R^2^ values obtained from 100 Y-randomization runs; the gray histogram and density curve represent the randomized results, while the red vertical reference line indicates the observed locked-test R^2^ of 0.5831. (**B**) Absolute prediction error versus maximum Tanimoto similarity; green points represent compounds inside the applicability domain, whereas red points represent compounds outside the domain. (**C**) Endpoint- and reactive-alert-specific R^2^ values; blue bars represent the endpoint-source subsets, and green bars represent the reactive-alert subsets. The frozen scaffold model was not modified following these analyses (Table 8).

The influence of potentially reactive compounds was evaluated without changing the frozen consensus model of the protein. Among the 934 locked-test compounds without any predefined reactive alerts, R^2^ was 0.5717. For the 104 compounds containing at least one alert, R^2^ was 0.5674, which was acceptable. Similar values indicated that the overall scaffold-test performance was not restricted to or predominantly driven by the potentially reactive subset. The endpoint- and reactive alert-specific results are summarized in Table 8 and displayed as part of the robustness analysis in Figure 3.

### 2.8. Y-Randomization Analysis

Y-randomization was conducted to determine whether the predictive signal could be reproduced after the original relationship between the molecular structure and pActivity was disrupted [26]. The dominant frozen ensemble component, ExtraTreesMorgan, was retrained using 100 independently permuted response vectors while preserving the molecular representation, development compounds, validation structure and model configuration.

The randomized models produced a mean R^2^ of −0.1233 with a standard deviation of 0.0495. The highest R^2^ observed across all 100 randomized runs was −0.0033, and none of the randomized models approached the positive performance of the corresponding nonrandomized model. The resulting empirical one-sided *p*-value was 0.0099. The complete R^2^ distribution obtained from the 100 response permutations is shown in Figure 3A, and the numerical results are summarized in Table 9.

### 2.9. Similarity-Based Applicability Domain

A similarity-based applicability domain was defined using the maximum Morgan Tanimoto similarity of each evaluated compound in the development set [42]. The corresponding distance was calculated as 1 − maximum similarity. The domain threshold (D* = 0.5867) was derived from the mean development nearest-neighbor distance plus three standard deviations.

Of the 1038 compounds in the locked scaffold test, 962 were located within the defined applicability domain, corresponding to a coverage of 92.68%. For these compounds, the frozen consensus model achieved R^2^ = 0.6041 and RMSE = 0.9268. The remaining 76 compounds were classified as outside the applicability domain and produced R^2^ = −0.0043. Thus, the largest reduction in predictive reliability was concentrated in a relatively small subset with the weakest structural support from the development data.

Similarity-stratified analysis demonstrated a progressive improvement in predictive performance as the test compounds became more similar to the development chemical space. The corresponding R^2^ values from the lowest to the highest similarity quartile were 0.2560, 0.4915, 0.7326, and 0.7486. The applicability domain threshold, compound-level similarity distribution, and performance across similarity regions are shown in Figure 3B. The coverage and predictive performance inside and outside the defined domain are presented in Table 9. An integrated summary of the primary locked test, endpoint-specific, reactive alert, and applicability domain results is provided in Table S7.

### 2.10. Model Interpretation and Structural Attribution

Interpretability analysis was performed separately for the principal components of the fitted consensus because the final predictions combined distinct molecular representations with nonlinear algorithms. ExtraTreesMorgan was the dominant component of the deployed ensemble, with a frozen model weight of 0.6258. TreeSHAP analysis of this component [43] identified Morgan bits 935, 82, 1416, 242, and 504 as the most influential fingerprint features according to the mean absolute SHAP magnitude. The additional highly ranked fingerprint features included bits 881, 573, 1048, 80, and 1028.. The global TreeSHAP ranking of the dominant ExtraTreesMorgan component is presented in Figure S8.

**Figure 4:**
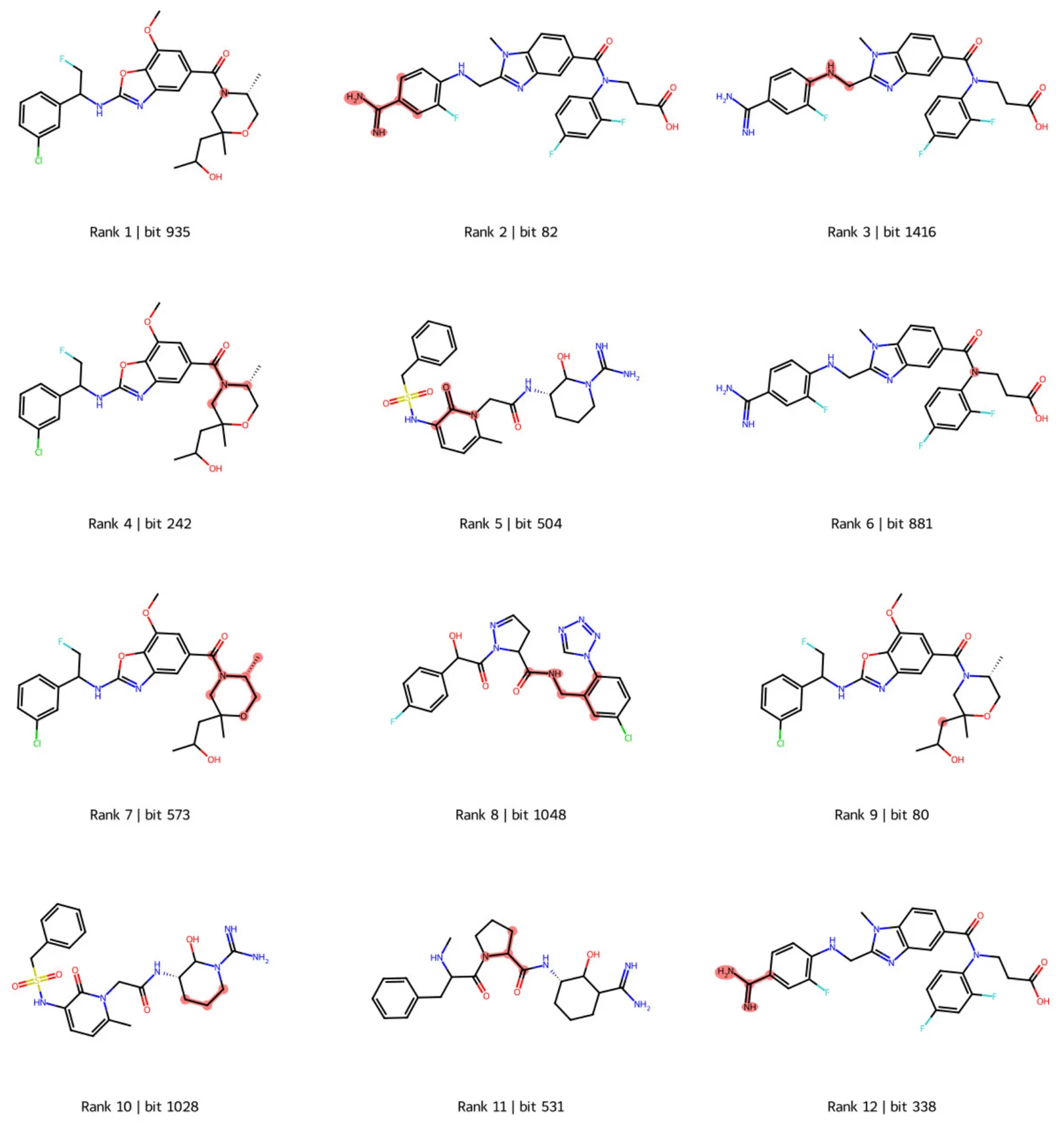
Representative molecular environments associated with influential Morgan fingerprint features identified by TreeSHAP analysis. Translucent red highlighting indicates the representative atom environment encoded by each bit, while conventional element colors are used for heteroatoms and halogens. The highlighted environments should not be interpreted as direct mechanistic evidence.

The influential Morgan bits were mapped back to the representative atom environments in molecules containing the corresponding fingerprint features. This procedure localizes the model attributions to defined molecular substructures rather than treating fingerprint-bit numbers as directly interpretable chemical variables. The highlighted environments include substituted aromatic and heteroaromatic systems, carbonyl-containing regions, nitrogen-containing motifs, and other local structural arrangements. Because hashed Morgan bits may represent more than one atomic environment, the displayed structures were treated as representative examples rather than unique definitions of each fingerprint feature. The representative atomic environments associated with the most influential Morgan bits are shown in Figure 4.

For the Mordred-based SVR component, permutation importance [44] calculated using the development data identified n7FARing, SlogP_VSA4, nG12AHRing, MINssCH2, SsssB, JGI5, NdsCH, Xch-6d, NdNH, and BCUTc-1h as the most influential descriptors. Collectively, these variables encoded information associated with ring topology, lipophilic surface area distribution, atom-state counts, molecular connectivity, and charge-related properties. The complete development-only permutation-importance ranking is presented in Figure S9.

Local SHAP values were used to distinguish between positive and negative contributions to individual predictions. The direction and magnitude of a feature’s contribution varied according to the complete molecular context and the remaining features present in the same compound. Consequently, a substructure associated with a positive contribution to one molecule was not automatically interpreted as an activity-enhancing group in every chemical series. Therefore, structural attributions were reported as model explanations and hypotheses for subsequent medicinal chemistry evaluations rather than as evidence of causal molecular mechanisms. Representative compounds with comparatively low, median, and high prediction errors are shown in Figure S10.

### 2.11. Supplementary Data and Reproducibility Records

The Supplementary Information was prepared to support transparent evaluation of the computational analyses. It contains the principal curation and partition summaries, feature-processing audit, selected hyperparameter configurations, model rankings, held-out and sensitivity metrics, and interpretation figures. The fixed project seed was 20260823, and the locked scaffold-test responses were evaluated only after completion of model selection and freezing of the final candidate.

## 3. Discussion

### 3.1. Predictive Performance and Selection of the ExtraTrees Model

In this study, we developed an assay-aware, multi-view, and explainable machine-learning framework for predicting thrombin-inhibitory activity from systematically curated ChEMBL records. The revised analysis was designed to evaluate the predictive performance within the represented chemical space while determining the extent to which the learned relationships could be transferred to compounds containing previously unseen molecular scaffolds. Accordingly, the model performance was examined using two complementary validation strategies: a conventional random held-out partition and a more stringent scaffold-disjoint locked test.

Under the random-split protocol, the development-selected ConsensusAll model achieved an out-of-fold R^2^ of 0.7588 and a held-out test R^2^ of 0.7480, with RMSE and MAE values of 0.7360 and 0.5307, respectively. The close agreement between the development out-of-fold and held-out statistics indicates that the random test performance was not obtained through direct optimization against the test responses. The revised random-test R^2^ also exceeded the value of 0.7246 obtained using the ExtraTrees model in the original analysis. This improvement was associated with an expanded and more systematically curated dataset, complementary use of Mordred descriptors and Morgan fingerprints, model-specific hyperparameter optimization, and cross-fitted consensus construction.

The scaffold-disjoint protocol addresses a substantially more demanding predictive problem. Complete Bemis–Murcko scaffold groups were separated between the development and locked test sets, resulting in 1180 development scaffolds and 295 test scaffolds with zero overlap [25]. Under these conditions, the frozen ConsensusTop5 model achieved R^2^ = 0.5831, RMSE = 0.9484, MAE = 0.7290, CCC = 0.7257, and Pearson’s *r* = 0.7693. Although the scaffold-test R^2^ was lower than the random held-out value, it remained close to the scaffold-grouped development out-of-fold R^2^ of 0.5983. This agreement indicates that the level of generalization observed after opening the locked test was consistent with that anticipated during the development.

The absolute difference of 0.1649 between the random held-out and scaffold-disjoint R^2^ values demonstrates the influence of structural partitioning on the apparent predictive performance. Random splitting can distribute close analogs or members of the same medicinal chemistry series across both the development and test subsets. Under these conditions, the model is evaluated partly on compounds containing structural patterns closely related to those encountered during training. Scaffold-disjoint validation removes overlap at the core framework level and, therefore, provides a more conservative assessment of the transfer to a new chemical series [25,39,45].

Consequently, the two validation strategies should be interpreted as addressing different but complementary questions. Random held-out performance estimates interpolation within or near the represented chemical space is relevant when candidate libraries contain analogs of known thrombin inhibitors. The scaffold-disjoint result more closely approximates lead-hopping or the prioritization of compounds with core frameworks absent from development. Therefore, the lower scaffold R^2^ should not be interpreted simply as model deterioration; rather, it quantifies the additional difficulty of new-scaffold generalization.

The bootstrap 95% confidence interval for the locked scaffold-test R^2^ ranged from 0.5424 to 0.6198, supporting the persistence of a positive predictive signal across the resampled test compositions. Nevertheless, the scaffold-test RMSE of approximately 0.95 pActivity units indicates that individual predictions may deviate substantially from experimental measurements. Therefore, the model is more suitable for compound ranking, prioritization, and hypothesis generation than for replacing biochemical potency determination.

An important methodological improvement over the original analysis was the complete separation of model development from the final scaffold testing. Descriptor filtering, missing value imputation, hyperparameter optimization, candidate ranking, and consensus construction were performed using the development data only. ConsensusTop5 was frozen before the locked-test responses were accessed, and no feature, parameter, model component, or ensemble configuration was modified after the final evaluation. This separation prevents test-set-driven model selection and strengthens the interpretation of the locked result as a genuinely held-out assessment [46].

### 3.2. Influence of Assay-Aware Curation and Replicate Handling

Public bioactivity repositories provide broad chemical coverage but contain records collected from different publications, laboratories, assay formats, and experimental conditions [16]. Without systematic curation, duplicate structures, inconsistent units, relational values, validity warnings, and repeated measurements may introduce avoidable response noise or data leakage [17,18,19]. Therefore, the revised workflow required exact activity relations, nanomolar units, valid standardized structures, and the absence of ChEMBL data-validity warnings before a record could enter the modeling dataset.

Replicate handling was particularly important because treating repeated measurements as independent compounds may artificially increase the dataset size and allow identical molecular structures to occur in multiple validation subsets. In the present workflow, records were grouped by standardized structure and endpoints before aggregation. Single measurements were retained directly, whereas replicate groups were summarized using their median only when the total pActivity span did not exceed 1.0 logarithmic unit. Ninety-six highly discordant structure–endpoint groups were then excluded.

Median aggregation was selected because it reduces sensitivity to isolated extreme measurements and does not require normally distributed replicates. This approach is more representative than retaining the first reported measurement or automatically selecting the most potent values. Choosing the minimum concentration for duplicate structures could introduce systematic potency bias, particularly when the measurements originate from assays with different levels of experimental uncertainty.

The presence of 96 conflicting replicate groups confirms that identical standardized structures do not invariably have interchangeable public-activity values. Such disagreements may arise from differences in substrate concentration, assay technology, buffer conditions, incubation time, inhibition mechanism, laboratory procedures, or reporting quality. Excluding highly discordant groups prevents the evaluation of a deterministic structure-based model against response values that cannot be consistently represented.

Nevertheless, replicate aggregation cannot eliminate all experimental uncertainties. Even concordant measurements may reflect assay-specific conditions, and the median represents a practical structure-level summary rather than a universal potency constant. Therefore, part of the remaining prediction error likely reflects experimental and inter-assay variability rather than the limitations of the molecular representations alone.

### 3.3. Integration of Ki and IC_50_ Measurements

The simultaneous availability of *K*i and IC_50_ records increased the chemical coverage of the final dataset but also introduced an important source of endpoint heterogeneity. Although both endpoints characterize inhibition, they do not represent the same physical quantities. *K*i is the inhibition constant associated with inhibitor binding under a defined kinetic model, whereas IC_50_ is the concentration that produces 50% inhibition under a specific experimental configuration. The relationship between the two depends on factors such as substrate concentration, substrate affinity, inhibition mechanism, incubation time, and assay format [20,21].

Accordingly, the pooled response used here should be interpreted as a combined logarithmic inhibitory activity measure rather than a homogeneous experimental p*K*i or pIC_50_ endpoint. The revised curation procedure reduced uncontrolled endpoint mixing by preserving the original endpoint identity, aggregating replicates separately within each endpoint, and evaluating cross-endpoint concordance before pooling the data. Structures represented by both *K*i and IC_50_ measurements were retained only when their median values differed by no more than 1.0 logarithmic unit. Forty-nine discordant cross-endpoint pairs were excluded using this rule.

Endpoint-specific locked-test analyses provided a direct assessment of heterogeneity. The frozen consensus model achieved R^2^ = 0.6053 for the 652 *K*i-only compounds and R^2^ = 0.5097 for the 369 IC_50_-only compounds. Endpoint-specific refitting of the dominant ExtraTreesMorgan component produced the same overall pattern, with R^2^ values of 0.6197 for *K*i and 0.4711 for IC_50_. The higher performance for *K*i is consistent with its more direct relationship with inhibitor binding and its comparatively lower dependence on assay-specific substrate conditions.

The positive IC_50_-specific performance nevertheless demonstrates that the pooled model did not derive its predictive signal exclusively from the *K*i records. The retention of both endpoints substantially expanded the chemical coverage and enabled the analysis of a broader inhibitor space. Therefore, endpoint-stratified metrics provide an appropriate balance: the pooled model supports wider compound prioritization, whereas the subgroup results indicate that predictions based on IC_50_-derived training information may carry greater uncertainty.

Only 17 locked test compounds contained concordant information from both endpoints, limiting the statistical interpretation of this subgroup. Future studies should ideally measure *K*i and IC_50_ for chemically diverse compounds under standardized conditions. Such data could support endpoint-specific calibration, hierarchical modeling, or multi-task learning, which explicitly separates common structural information from endpoint-dependent effects.

### 3.4. Potentially Reactive and Time-Dependent Inhibitors

Some thrombin inhibitors contain electrophilic functional groups that may support reversible, irreversible, or time-dependent inhibition [34]. If such structures dominate a dataset, a model could partially associate activity with the presence of reactive motifs rather than with the broader molecular determinants of thrombin recognition. Therefore, the revised workflow screened the curated compounds for boronic acids or esters, activated alkenes, α-ketoamides, nitriles, acrylamides, aldehydes, vinyl sulfones, and sulfonyl fluorides.

At least one alert was identified in 520 compounds in this study. However, the presence of a potentially reactive substructure does not indicate a covalent mechanism. Reactivity depends on the electronic and steric environment of the functional group, orientation within the binding site, accessibility of target nucleophiles, exposure time, and experimental conditions. Therefore, the alerted compounds were not automatically excluded from the primary dataset.

Instead, the reactive alert status was retained as a sensitivity variable. In the locked scaffold test, compounds without alerts achieved R^2^ = 0.5717, whereas alerted compounds achieved R^2^ = 0.5674. Similar results indicate that the overall predictive performance was not restricted to or predominantly driven by potentially reactive chemotypes.

Nevertheless, this analysis should be considered a structural robustness assessment rather than a mechanistic classification. The available ChEMBL records generally lack the kinetic measurements required to distinguish reversible inhibition from covalent or time-dependent mechanisms. Reliable mechanistic assignment requires preincubation studies, reversibility experiments, measurements of *k*inact and *K*I, mass spectrometric evidence, or structural confirmation of covalent binding. Mechanistically annotated datasets may permit the separate modeling of reversible and covalent thrombin inhibitors.

### 3.5. Complementary Molecular Representations and Consensus Modeling

Mordred descriptors and Morgan fingerprints were used because they encode complementary molecular information. Mordred descriptors summarize the global constitutional, topological, physicochemical, electronic, and surface area properties [35]. Morgan fingerprints encode local circular atom environments and are particularly effective in recognizing recurring structural motifs within medicinal chemistry series [24].

ExtraTreesMorgan was the strongest individual candidate under both validation strategies. It achieved development of out-of-fold R^2^ = 0.7448 in the random analysis and R^2^ = 0.5714 under scaffold-grouped validation. Its stronger performance in the random setting is consistent with the ability of circular fingerprints to recognize local environments that are shared among structurally related compounds. Nevertheless, its retention as the strongest single scaffold candidate indicates that fingerprint features also contain transferable information beyond complete scaffold identity.

Descriptor-based and combined models contributed to complementary predictive patterns. XGBoostCombined and LightGBMCombined modeled nonlinear interactions between global descriptors and local fingerprint features, whereas SVRMordred captured continuous relationships in the descriptor space. Although these models did not individually outperform ExtraTreesMorgan, their partially different errors contributed to the improvement obtained through consensus modeling.

ConsensusAll increased the random development out-of-fold R^2^ from 0.7448 for the strongest individual model to 0.7588. Similarly, ConsensusTop5 increased the scaffold-grouped R^2^ from 0.5714 to 0.5983. These improvements were moderate but consistent across all the experiments. The non-negative weighting strategy prevented unstable cancellation between the model predictions, whereas cross-fitting ensured that the consensus weights were estimated from the predictions generated for compounds not used to fit the corresponding base model [40].

Regression was preferred over classification because the available thrombin activities constituted a continuous response that spanned several logarithmic units. Converting these measurements into active and inactive categories would require an arbitrary threshold, discard differences in potency within each class, and potentially assign nearly identical compounds to the opposing categories. Continuous pActivity prediction is therefore better aligned with compound ranking and lead optimization than other methods.

### 3.6. Y-Randomization and Evidence Against Chance Correlation

Y-randomization was expanded to 100 independent response permutations in the revised analyses. The randomized ExtraTreesMorgan models produced a mean R^2^ of −0.1233, with a maximum R^2^ of −0.0033. None approached the positive performance of the non-randomized model, and the empirical *p*-value was 0.0099. These results indicate that comparable predictive performance was not achieved after the disruption of the original relationship between the molecular structure and activity [26].

The negative randomized R^2^ values demonstrate that the models trained on permuted responses performed worse than a simple mean-response predictor. This supports the conclusion that the non-randomized workflow captured systematic structure–activity information rather than a relationship that could be readily reproduced by chance.

Successful Y-randomization alone does not establish external predictability. A model can fail to predict randomized responses while remaining affected by structural redundancy, assay heterogeneity or limited chemical space coverage. Y-randomization was therefore treated as a complementary negative control rather than a substitute for out-of-fold validation, held-out testing, or scaffold-disjoint evaluation. Its value lies in strengthening the combined validation evidence, rather than independently proving model generalizability.

### 3.7. Applicability Domain and Similarity-Dependent Reliability

The applicability domain was defined using the nearest-neighbor Morgan Tanimoto similarity because this measure directly quantifies the structural support available for each prediction. The previous Williams plot was based on molecular leverage and the standardized residuals. Although Williams plots are useful for identifying influential observations, they do not replace structure-aware validation and may be difficult to align with nonlinear models using fingerprint representations [27,45,46,47].

The similarity-based domain covered 962 of the 1038 locked scaffold-test compounds, corresponding to 92.68% of the test set size. Within this domain, R^2^ increased from the overall scaffold-test value of 0.5831 to 0.6041, and the RMSE decreased to 0.9268. In contrast, the 76 compounds outside the domain produced R^2^ = −0.0043. This marked difference demonstrates that predictive reliability depends strongly on the presence of structurally related local environments in the development set, even when complete Bemis–Murcko scaffolds remain disjoint.

The similarity quartile analysis provided further evidence for this relationship. R^2^ increased from 0.2560 in the lowest-similarity quartile to 0.4915, 0.7326, and 0.7486 across progressively more similar compounds, respectively. Thus, the model achieved strong performance for some scaffold-disjoint compounds when their local molecular environments were represented, whereas predictions for the most structurally distant compounds were substantially less reliable.

These findings have practical implications for prospective applications. Predicted pActivity values should be accompanied by a domain or similarity indicators. Compounds inside the domain may be prioritized with greater confidence, whereas outside-domain predictions should be regarded as exploratory analyses. The experimental evaluation of structurally distant compounds would also provide informative data for expanding the supported chemical space during future model updates.

The applicability domain results help explain the difference between the random and scaffold-disjoint performances. Random partitioning increases the probability that each test compound has a close developmental analog. Scaffold separation reduces this support, particularly in the low-similarity region. Therefore, the validation gap reflects a measurable reduction in structural information rather than a change in statistical sampling.

### 3.8. Structural Interpretation and Chemical Relevance

Interpretability analysis was performed separately for the principal consensus components because the final predictions combined different algorithms and molecular representations. TreeSHAP analysis of the dominant ExtraTreesMorgan component identified Morgan bits 935, 82, 1416, 242, and 504 as the most influential fingerprint features. Mapping these bits to representative atomic environments connects their numerical identifiers with localized molecular substructures.

The highlighted environments include substituted aromatic or heteroaromatic systems, nitrogen-containing motifs, carbonyl-associated regions, and other local arrangements of atoms. These patterns are chemically plausible because thrombin recognition depends on an appropriate combination of hydrogen bonding, hydrophobic interactions, charge distribution, molecular topology, and the orientation of substituents. However, hashed fingerprint bits can represent more than one atomic environment, and the contribution of a motif depends on the remainder of the molecule. Therefore, the displayed structures should be regarded as representative environments rather than uniquely defined pharmacophores.

Permutation-importance analysis of the SVRMordred component identified n7FARing, SlogP_VSA4, nG12AHRing, MINssCH2, SsssB, JGI5, NdsCH, Xch-6d, NdNH, and BCUTc-1h as the influential global descriptors. Collectively, these variables encode information related to the ring topology, lipophilic surface area distribution, atom-state counts, molecular connectivity, and charge-associated properties. Their importance suggests that thrombin potency depends on a coordinated balance of topological, hydrophobic, and polar characteristics, rather than on a single molecular property.

The sign of a local SHAP contribution is specific to individual predictions. A substructure associated with a positive contribution in one molecule may have a smaller or negative contribution in another molecule because of differences in substituents, feature combinations, and molecular context. Similarly, the global mean absolute SHAP values rank the magnitude of feature influence but do not provide a universally favorable direction [28,48]. Consequently, the present study avoids describing isolated fragments as invariably activity-enhancing or activity reducing.

SHAP values and permutation importance explain the behavior of a fitted model; however, they do not establish molecular mechanisms or causality [24,48]. Correlated features may share attribution, and the model may use a structural pattern as a marker for a chemical series rather than because the pattern directly interacts with thrombin, for example. Therefore, structural interpretations are most appropriately used to generate hypotheses, guide analog comparisons, and prioritize compounds for experimental evaluation.

### 3.9. Comparison with Previous Explainable Modeling of Thrombin Inhibitors

Masand et al. developed an explainable GA-XGBoost model using 2803 thrombin inhibitors with definite *K*i measurements [32]. Their final model incorporated eight molecular descriptors selected from a large three-dimensional descriptor pool and was evaluated using leave-10–out cross-validation and a randomly generated 80:20 training–prediction partition. The study reported strong statistical performance and employed SHAP and ceteris paribus analyses to identify nitrogen-containing environments, hydrogen-bonding arrangements, sp^3^ carbon atoms, and amide-related characteristics as important determinants of thrombin inhibition.

Direct numerical comparison with the present results is not appropriate because the studies differ in endpoint definition, dataset composition, molecular representation, and validation design. Masand et al. modeled a homogeneous p*K*i endpoint using PM3-optimized three-dimensional descriptors, whereas the present study integrated carefully harmonized *K*i and IC_50_ measurements and combined two-dimensional Mordred descriptors with Morgan fingerprints. Moreover, their Q^2^F statistics for a randomly generated prediction set are not numerically interchangeable with either the conventional held-out R^2^ or the scaffold-disjoint locked-test R^2^ reported herein.

The present study complements the work of Masand et al. by focusing on assay-aware replicate and endpoint harmonization, sensitivity analysis of potentially reactive compounds, development-only model selection, and explicit separation of random interpolation from new-scaffold generalization. Additional contributions include a one-time scaffold-disjoint locked test, 100-run Y-randomization, similarity-stratified applicability domain assessment, bootstrap uncertainty intervals, and comprehensive machine-readable Supplementary Data. Accordingly, the novelty of the present study lies in its integrated validation and reproducibility framework, rather than in the first application of machine learning or SHAP to thrombin inhibitors.

The descriptor-level interpretations of the two studies are nevertheless broadly complementary. Masand et al. emphasized ring and non-ring nitrogen environments, hydrogen-bond donor and acceptor arrangements, sp^3^ carbon atoms, and amide-related features [32]. The Mordred analysis similarly identified ring-, nitrogen-, topology-, and surface area-related variables among the influential descriptors. However, neither study supports a simple rule according to which increasing the nitrogen content or adding a particular fragment invariably increases thrombin potency. These effects remain dependent on the molecular context, protonation state, topology, and relative positioning of complementary functional groups. This context dependence is consistent with the subsequent analysis of nitrogen-containing thrombin inhibitors reported by Masand et al. [33].

### 3.10. Reproducibility and Prospective Utility

The revised workflow was designed to preserve the principal data and analytical records required to evaluate the reported results. The accompanying Supplementary Data include curated compound-level data, endpoint labels, replicate classifications, scaffold assignments, development and locked-test manifests, predefined cross-validation folds, feature schemas, development-derived imputation values, hyperparameter search records, selected configurations, out-of-fold and held-out predictions, robustness analysis outputs, software information, and random seeds. These materials enable the independent examination of curation, partitioning, feature processing, model selection, and validation procedures.

The most appropriate prospective application of the developed model is the prioritization of candidate compounds before biochemical testing. Within the chemical space represented by the training data, the reported relationships may support virtual library ranking, analog selection, and reduction in the number of compounds advanced to experimental assays. Predictions for compounds within the applicability domain should be regarded as having strong structural support, whereas predictions for structurally dissimilar compounds should be interpreted cautiously.

The model is not intended for clinical decision-making or as a replacement for experimental pharmacology. Predicted thrombin potency alone does not establish target selectivity, anticoagulant efficacy, pharmacokinetic suitability, metabolic stability, or acceptable bleeding-risk profile. Therefore, model-based prioritization should be followed by biochemical potency measurements, selectivity profiling, absorption, distribution, metabolism, excretion, toxicity assessments, and, where appropriate, structure-based analyses. Prospective testing of newly synthesized compounds is required to establish the practical utility and broader generalizability of the framework.

### 3.11. Limitations and Future Directions

This study has several limitations. First, this was a retrospective study based on records obtained from a single public bioactivity repository. Although ChEMBL integrates measurements from numerous publications and assays, this diversity does not constitute a prospective external validation. A genuinely external assessment should include compounds from a later temporal release, a separate data source, or newly synthesized candidates tested after the modeling workflow is frozen [45].

Second, assay-aware curation reduces but cannot completely eliminate experimental heterogeneity. Measurements reported using the same endpoint and unit may originate from different substrate concentrations, assay technologies, incubation times, buffer conditions, and kinetic assumptions. Some assay metadata were incomplete or inconsistently represented in the source records. Consequently, part of the remaining prediction error may reflect experimental variability that cannot be inferred from the two-dimensional molecular structure.

Third, the pooled pActivity response does not represent a single, homogeneous biophysical endpoint. Endpoint-specific sensitivity analyses partly address this limitation; however, separate *K*i and IC_50_ models remain constrained by reduced sample sizes and differences in chemical space composition. Larger standardized datasets could support multitask or hierarchical models that distinguish endpoint-specific effects from shared molecular information.

Fourth, structural alert analysis cannot definitively distinguish between covalent, reversible covalent, irreversible, or time-dependent inhibition. Kinetic and structural experiments are required for a mechanistic assignment. The similar performance observed for alerted and non-alerted compounds demonstrates robustness but does not establish identical inhibition mechanisms.

Fifth, scaffold-disjoint validation remains an approximation of prospective generalizations. Bemis–Murcko scaffolds separate core molecular frameworks but do not completely characterize chemical similarities. Compounds with different scaffolds may retain related local pharmacophoric environments, whereas small modifications within the same scaffold can sometimes produce substantial changes in activity. The similarity-stratified results demonstrate that structural support is continuous rather than fully represented by a binary shared/unshared scaffold designation.

Sixth, the model interpretation is limited by correlated descriptors, hashed fingerprint collisions, and context-dependent SHAP contributions. Important descriptors and fingerprint environments identify the information used by the model but should not be interpreted as isolated causal determinants of thrombin inhibition.

Finally, the present study did not include prospective synthesis or biochemical evaluations of the newly prioritized compounds. Future studies should apply the frozen workflow to an external compound collection, select candidates without access to their experimental activities, and evaluate them using standardized thrombin assays. A later ChEMBL release could provide an additional temporal validation. Reporting all prospective outcomes, including unsuccessful predictions, is essential for obtaining an unbiased estimate of real-world performance.

Despite these limitations, the revised analysis substantially strengthens the evidence supporting the proposed QSAR model. The random held-out evaluation demonstrated useful predictive performance within the represented chemical space, whereas the locked scaffold test provided a conservative assessment of new-core generalization. Endpoint-specific, reactive alert, Y-randomization, and applicability domain analyses clarified the conditions under which predictions were supported. Together with the accompanying Supplementary Information, these findings provide a transparent foundation for hypothesis generation and experimental prioritization in thrombin-inhibitor discovery.

## 4. Materials and Methods

### 4.1. Data Source, Molecular Standardization, and Endpoint Definition

Bioactivity records for human thrombin were retrieved from the ChEMBL database on 10 August 2026 using the target identifier CHEMBL204 [16]. The initial dataset contained 9327 activity records before curation. The retrieved table includes molecular identifiers, canonical SMILES representations, activity types, standard activity values, relational qualifiers, concentration units, assay annotations, and ChEMBL data-validity comments.

Only measurements reported as inhibition constants (Ki) or half-maximal inhibitory concentrations (IC_50_) were considered. Records were required to have a numerical and positive standard activity value, an exact standard relation (“=”), and a standard concentration unit of nanomolar (nM). Studies that reported measurements using inequality relations, approximate values, percentages, or nonconvertible units were excluded. Records containing ChEMBL data validity warnings were also removed before molecular processing.

The predefined curation workflow reduced the initial 9327 records to 6849 exact-relation entries, 6777 nanomolar measurements, and 6435 valid records. All filtering decisions and record-level exclusion reasons were preserved in the curation audit supplied in the Supplementary Data.

The molecular structures were parsed and processed using RDKit [49]. The available SMILES strings were converted to RDKit molecular objects and subjected to sanitization. Structures that could not be parsed or chemically sanitized were excluded from the study. Molecular standardization reduces representational inconsistencies caused by alternative aromaticity assignments, disconnected counterions, salts, and non-canonical SMILES encodings [17,18,19].

The standardized canonical SMILES representation was used as the principal structure identifier for duplicate detection and subsequent compound-level operations. Records with the same standardized structure and identical activity information were treated as exact duplicates, and only one copy was retained. This procedure reduced the structurally valid set from 6434 to 6414 eligible measurements points. Standardization was completed before partitioning, descriptor calculation, scaffold generation and fingerprint construction.

All retained Ki and IC_50_ measurements were expressed in nanomolar units. Each measurement was transformed into its corresponding negative logarithmic activity using Equation (1).

(1) $$pActivity=9-{log}_{10}\;\left(Activity\;\left[nM\right]\right)$$

This transformation is equivalent to −log_10_(Activity [M]), and consequently, higher pActivity values indicate greater experimentally reported inhibitory potency. Values outside the predefined interval of 3.0–12.0 were excluded from the analysis. Because the final response contained both Ki- and IC_50_-derived values, it was designated as pActivity rather than pKi or pIC_50_ [20,21]. The original endpoint identity was retained for every record and compound. Regression was selected because the measurements formed a continuous response spanning several logarithmic units; conversion to categorical activity classes would require an arbitrary threshold and discard quantitative potency data.

### 4.2. Replicate Aggregation and Cross-Endpoint Harmonization

Eligible measurements were first grouped according to the standardized molecular structure and original endpoint type. Groups containing a single measurement were retained. For groups containing two or more measurements, the replicate concordance was evaluated using Equation (2):

(2) $$\Delta_{rep}=max\left(pActivity\right)-min\left(pActivity\right)$$

Groups with Δrep ≤ 0.5 were classified as highly consistent, whereas groups with 0.5 < Δrep ≤ 1.0 were classified as acceptable groups. For both retained categories, the median pActivity was used as the structure–activity relationship. Groups with Δrep > 1.0 were classified as conflicting and excluded from the analysis.

Following endpoint-level aggregation, structures represented by both Ki and IC_50_ measurements were evaluated for cross-endpoint agreement using Equation (3).

(3) $$\Delta_{endpoint}=\left|median\left(pK_{i}\right)-median\left(pIC_{50}\right)\right|$$

Structures with Δendpoint ≤ 1.0 were retained and represented by the median of the concordant endpoint-level values. Structures that exceeded this boundary were excluded. This procedure identified 4843 single-measurement groups, 451 highly consistent groups, 80 acceptable groups, and 96 conflicting groups of patients. Forty-nine discordant cross-endpoint pairs were excluded, and the final dataset contained 5189 unique structures.

### 4.3. Identification of Potentially Reactive Substructures

Potentially reactive motifs were identified using predefined SMARTS patterns. The alerts investigated included boronic acids or esters, activated alkenes or Michael acceptors, α-ketoamides, nitriles, acrylamides, aldehydes, vinyl sulfones and sulfonyl fluorides [34]. A compound was considered alert-positive when at least one pattern matched its standardized structure. Multiple alerts can be assigned to a compound. The alert status was retained as metadata and was not used as a feature during model fitting. Alerted compounds were not automatically excluded because a structural motif alone does not establish covalent or time-dependent inhibition; instead, the alert status was used for predefined sensitivity analyses.

### 4.4. Random and Scaffold-Disjoint Dataset Partitioning

Two complementary partitioning strategies were employed in this study. First, the complete dataset was randomly divided into development and held-out subsets using an 80:20 ratio and a fixed project seed of 20260823. The development subset contained 4151 compounds, and the random held-out test contained 1038 compounds.

Second, a scaffold-disjoint partition was generated to evaluate the generalization of unseen core frameworks. Bemis–Murcko scaffolds were calculated from standardized structures using the RDKit [25,49]. Compounds sharing the same scaffold were treated as distinct groups. Exact mixed-integer linear programming minimized deviations from the desired 80:20 ratio while balancing the pActivity, endpoint composition, and reactive alert prevalence. The hard constraints prohibit scaffold overlap. The final scaffold development set contained 4151 compounds from 1180 scaffolds, and the locked test contained 1038 compounds from 295 scaffolds, with no overlap.

Five scaffold-grouped folds were defined within the development, with each scaffold confined to one fold. Locked responses were not accessed during feature preprocessing, hyperparameter optimization, candidate ranking, consensus construction, or selection of the final scaffold candidate.

### 4.5. Molecular Representations and Leakage-Safe Preprocessing

Two-dimensional molecular descriptors were calculated using Mordred, with three-dimensional descriptor classes disabled [35]. Mordred initially generated 1613 descriptors. All preprocessing operations were fitted using only the relevant development subset. Descriptors with more than 20% missing or non-finite development values were removed, retaining 1450 variables. Variance filtering retained 1312 descriptors. When two development descriptors exhibited an absolute Pearson correlation greater than 0.95, one member of the pair was removed according to the deterministic feature order, leaving 615 descriptors.

Missing values were replaced using the development medians. The selected descriptor names, removal decisions, and imputation values were frozen and applied unchanged to the held-out compounds. For algorithms requiring scaling, standardization parameters were fitted within the corresponding training data or cross-validation fold and transferred to the validation data.

Morgan fingerprints were generated from standardized RDKit molecules using a radius of 2, 2048 bits, and chirality. Bits constant across development were removed, leaving 2039 non-constant features. The selected bit indices and column order were frozen and applied unchanged to the held-out compounds. Morgan fingerprints were independently evaluated and concatenated with Mordred descriptors to create a combined feature view.

### 4.6. Regression Algorithms, Hyperparameter Optimization, and Consensus Modeling

The benchmark included ridge regression, ElasticNet, partial least-squares regression [39], support-vector regression, Nu-support-vector regression, random forest [44], extremely randomized trees [36], histogram gradient boosting, XGBoost [37], LightGBM [38], and CatBoost [50]. Candidate algorithms were evaluated using Mordred, Morgan, or a combination of feature views [51].

The hyperparameters were optimized independently for each candidate using only the development data. Random development folds were used for random analysis, whereas predefined scaffold-grouped folds were used for scaffold analysis. The mean fold RMSE was the primary optimization objective. The search spaces included model-appropriate tree counts and depths, split and leaf constraints, feature fractions, learning rates, subsampling fractions, regularization terms, kernel parameters, latent-component numbers, and support vector penalties. The selected hyperparameter configurations are summarized in Table S6 of the Supplementary Information.

Each development compound received an out-of-fold prediction from a model that was not trained using that compound. Candidate ranking was based on complete development out-of-fold predictions rather than training fit or held-out performance.

Consensus models were constructed from optimized candidate out-of-fold predictions using non-negative least squares regression [40]. Individual weights were constrained to be non-negative and normalized to sum to one. The ConsensusTop3, ConsensusTop5, and ConsensusAll configurations were evaluated. Cross-fitting prevented in-sample base model predictions from contributing to the ensemble-weight estimation. ConsensusAll was selected for random held-out evaluation, whereas ConsensusTop5 was selected for scaffold-disjoint testing. Each candidate and its weights were frozen before the associated held-out responses were accessed.

### 4.7. Predictive Performance Metrics and Confidence Intervals

The model performance was quantified using R^2^, RMSE, MAE, Pearson’s correlation, and Lin’s concordance correlation coefficient (CCC) [52]. R^2^ is calculated using Equation (4):

(4) $$R^{2}=1-\frac{\sum_{i=1}^{n}{\left(y_{i}-{\hat{y}}_{i}\right)}^{2}}{\sum_{i=1}^{n}{\left(y_{i}-\bar{y}\right)}^{2}}$$

The RMSE and MAE were calculated using Equations (5) and (6), respectively:

(5) $$RMSE=\sqrt{\frac{1}{n}\sum_{i=1}^{n}{\left(y_{i}-{\hat{y}}_{i}\right)}^{2}}$$

(6) $$MAE=\frac{1}{n}\sum_{i=1}^{n}\left|y_{i}-{\hat{y}}_{i}\right|$$

The CCC was calculated using Equation (7).

(7) $$CCC=\frac{2\rho \;\sigma_{y}\sigma_{\hat{y}}}{\sigma_{y}^{2}+\sigma_{\hat{y}}^{2}+{\left(\mu_{y}-\mu_{\hat{y}}\right)}^{2}}$$

where yi and ŷi are the experimental and predicted pActivity values, respectively; ȳ is the experimental mean; n is the number of evaluated compounds; ρ is the Pearson correlation; σ denotes the corresponding standard deviation; and μ denotes the corresponding mean. The development metrics were calculated from the pooled out-of-fold predictions. The notation Q^2^ was reserved for compounds excluded from the fitting and was not used for the training-set fit [39,46].

The uncertainty in the held-out metrics was quantified by nonparametric bootstrap resampling of paired experimental and predicted values [41]. The locked scaffold test used 2000 bootstrap replicates. The 95% confidence intervals were obtained from the 2.5th and 97.5th percentiles of each metric distribution. The fitted model was not retrained during this procedure; therefore, the intervals described the uncertainty associated with the finite test-set composition.

### 4.8. Endpoint- and Reactive-Substructure Sensitivity Analyses

Frozen locked-test predictions were stratified as Ki-only, IC_50_-only, concordant dual-endpoint, reactive-alert-positive, or reactive-alert-negative. The metrics were recalculated within each subgroup without modifying the fitted consensus. Complementary endpoint-specific ExtraTreesMorgan models were fitted using the available Ki and IC_50_ subsets while retaining structure-aware validation. These models were used only for sensitivity analysis and did not replace the primary consensus model.

### 4.9. Y-Randomization Analysis

Y-randomization was used to assess whether comparable performance could arise after the disruption of the structure–activity relationship [26]. The response vector was independently permuted 100 times while preserving the features, compound assignments, validation structure, and model configuration. For each permutation, the dominant frozen ExtraTreesMorgan component was retrained using the same data. The empirical one-sided *p*-value is calculated using Equation (8).

(8) $$p_{empirical}=\frac{1+\sum_{j=1}^{B}I\;\left(R_{random,j}^{2}\geq R_{observed}^{2}\right)}{B+1},\;B=100$$

Here, I is the indicator function, and B is the number of permutations. Adding one to the numerator and denominator prevents an empirical probability of zero.

### 4.10. Similarity-Based Applicability Domain

The applicability domain used Morgan Tanimoto similarity between each evaluated compound and the developed reference set [42]. For compound i, the structural distance is defined using Equation (9):

(9) $$D_{i}=1-\underset{j}{max}\left[T\left(i,j\right)\right]$$

The domain threshold was derived from the development of the nearest-neighbor distances using Equation (10):

(10) $$D*={\bar{D}}_{development}+3\;SD\;\left(D_{development}\right)$$

A test compound was inside the domain when Di ≤ D*, and outside the domain when Di > D*. This procedure produced a D* of 0.5867. The performance was calculated separately inside and outside the domain. Locked-test compounds were also divided into quartiles according to their maximum development similarity to examine similarity-dependent reliability [23,47].

### 4.11. Model Interpretation

TreeSHAP was applied to the dominant ExtraTreesMorgan component [43]. The global importance was summarized using the mean absolute SHAP values. Influential fingerprint bits were mapped to representative RDKit atom environments because hashing can assign multiple environments to one bit, and the displayed structures were treated as representative examples. For the Mordred-based SVR component, the development-only permutation importance quantified the reduction in performance after independent feature permutation [44]. Local SHAP values were interpreted as compound-specific contributions relative to the model baseline, and not as universal activity effects or causal mechanisms [24,48].

### 4.12. Reproducibility and Supplementary Data

The fixed project seed was 20260823. The recorded computational environment used Python 3.13.15 on 64-bit Linux. Recorded package versions included RDKit 2026.03.5, NumPy 2.1.3, pandas 2.2.3, and Optuna 4.9.0. RDKit was used for structure processing, scaffold generation, fingerprint calculation, and structural visualization [49]. Mordred was used to calculate the two-dimensional molecular descriptors [35]. Scikit-learn [51], XGBoost [37], LightGBM [38], and CatBoost [50] were used for model development. SHAP was employed for tree-model interpretation [28]. The Supplementary Information contains the principal curation and partition summaries, feature-processing audit, selected hyperparameter configurations, validation metrics, sensitivity analyses, and interpretation figures. The Supplementary Information includes curation-audit tables, scaffold-partition summaries, feature-filtering results, development-only model rankings, selected hyperparameter configurations, held-out and sensitivity metrics, and interpretation figures. These records support transparent examination of the principal data-curation, feature-processing, model-selection, and validation decisions reported in this study.

## 5. Conclusions

This study established an assay-aware, explainable, and leakage-safe machine-learning QSAR framework for predicting thrombin-inhibitory activity. The systematic curation of ChEMBL records produced a dataset of 5189 unique compounds after molecular standardization, replicate-concordance assessment, cross-endpoint harmonization, and exclusion of conflicting measurements. The combined use of two-dimensional Mordred descriptors and Morgan fingerprints enabled the incorporation of complementary global and substructural information into the modeling workflow.

Under conventional random held-out evaluation, the development-selected ConsensusAll model achieved R^2^ = 0.7480, RMSE = 0.7360, MAE = 0.5307, and CCC = 0.8540. The close agreement with the development of out-of-fold R^2^ of 0.7588 demonstrated a stable predictive performance within the represented chemical space. Under the more stringent scaffold-disjoint protocol, the frozen ConsensusTop5 model retained R^2^ = 0.5831, RMSE = 0.9484, MAE = 0.7290, and CCC = 0.7257 across 1038 compounds representing 295 scaffolds absent from model development. The difference between the random and scaffold-disjoint performances highlights the optimistic estimates that may arise when structurally related compounds are distributed across randomly generated subsets.

Endpoint-specific analyses demonstrated predictive signals for both Ki- and IC_50_-derived activities, although the performance was higher for the Ki-only subset. The comparable performance between compounds with and without potentially reactive structural alerts indicated that the overall result was not driven exclusively by electrophilic chemotypes. One hundred Y-randomization runs failed to reproduce the observed predictive signal, while the similarity-based applicability domain covered 92.68% of the locked scaffold test and increased R^2^ to 0.6041 for structurally related compounds.

TreeSHAP, fingerprint-environment mapping, and Mordred permutation importance provided complementary structural explanations for the model predictions. These attributions identify the molecular information used by the fitted models but should not be interpreted as direct evidence of causal binding mechanisms. The supplementary feature-processing audit, selected hyperparameter configurations, validation summaries, and applicability-domain results provide a transparent basis for evaluating the scope of the reported analyses.

Overall, the framework provides useful quantitative prioritization within the represented thrombin-inhibitor space and retains a measurable predictive capability for previously unseen scaffolds. Nevertheless, it should be regarded as a hypothesis-generating tool rather than a replacement for experimental potency determination. Prospective evaluation using newly synthesized compounds, standardized thrombin assays, and temporally or externally independent datasets is necessary to establish broader generalizability and practical value in thrombin-inhibitor discovery.

## Figures and Tables

**Table 1:** Summary of assay-aware curation and final thrombin-inhibitor dataset.

Parameter	Value
Initial ChEMBL activity records	9327
Records with exact activity relation	6849
Records reported in nM	6777
Records without validity warnings	6435
Structurally valid records	6434
Eligible records after exact-duplicate removal	6414
Eligible Ki records/unique structures	3988/3445
Eligible IC_50_ records/unique structures	2426/2025
Single/highly consistent/acceptable groups	4843/451/80
Conflicting replicate groups excluded	96
Conflicting cross-endpoint pairs excluded	49
Ki-only/IC_50_-only/both	3259/1843/87
Final unique compounds	5189
Reactive-alert/no-alert compounds	520/4669

**Table 2:** Summary of molecular representation and feature preprocessing.

Representation	Processing Stage	Features
Mordred	Initially calculated 2D descriptors	1613
Mordred	Missingness ≤ 20%	1450
Mordred	Variance filtering	1312
Mordred	Correlation filtering (|r| ≤ 0.95)	615
Morgan	Initial radius-2 fingerprint	2048
Morgan	Nonconstant development bits	2039

**Table 3:** Development of out-of-fold ranking in random-split analysis.

Rank	Candidate	Representation	R^2^	RMSE	MAE	CCC
1	ConsensusAll	Consensus	0.7588	0.7234	0.5334	0.8571
2	ConsensusTop3	Consensus	0.7510	0.7349	0.5422	0.8519
3	ExtraTreesMorgan	Morgan	0.7448	0.7440	0.5435	0.8527
4	XGBoostCombined	Combined	0.7170	0.7835	0.5894	0.8236
5	LightGBMCombined	Combined	0.7080	0.7958	0.6033	0.8208
6	SVRMordred	Mordred	0.6931	0.8159	0.6091	0.8241
7	NuSVRMorgan	Morgan	0.6406	0.8829	0.6702	0.7772

**Table 4:** Predictive performance of ConsensusAll in the random-split analysis.

Dataset	n	R^2^	RMSE	MAE	CCC	Pearson r
Development OOF	4151	0.7588	0.7234	0.5334	0.8571	0.8724
Random held-out test	1038	0.7480	0.7360	0.5307	0.8540	0.8653

**Table 5:** Composition of the scaffold-disjoint development and locked test sets.

Parameter	Development	Locked Test
Compounds	4151	1038
Bemis–Murcko scaffolds	1180	295
Mean pActivity	6.5613	6.5598
SD pActivity	1.4723	1.4697
Ki-only	2607	652
IC_50_-only	1474	369
Both endpoints	70	17
Reactive alerts	416	104
Shared scaffolds	0	0

**Table 6:** Development of out-of-fold ranking under scaffold-grouped validation.

Rank	Candidate	Representation	R^2^	RMSE	MAE	CCC
1	ConsensusTop5	Consensus	0.5983	0.9331	0.7230	0.7268
2	ConsensusAll	Consensus	0.5975	0.9340	0.7238	0.7264
3	ConsensusTop3	Consensus	0.5834	0.9502	0.7332	0.7205
4	ExtraTreesMorgan	Morgan	0.5714	0.9637	0.7330	0.7245
5	XGBoostCombined	Combined	0.5447	0.9933	0.7767	0.6891
6	LightGBMCombined	Combined	0.5369	1.0018	0.7834	0.6789
7	NuSVRMorgan	Morgan	0.5204	1.0195	0.7946	0.6810

**Table 7:** Final performance of ConsensusTop5 under scaffold-disjoint validation.

Dataset	n	R^2^	RMSE	MAE	CCC	Pearson r
Scaffold-grouped development OOF	4151	0.5983	0.9331	0.7230	0.7268	0.7845
Locked scaffold test	1038	0.5831	0.9484	0.7290	0.7257	0.7693

**Table 8:** Endpoint- and reactive-alert-specific performance in the locked-scaffold test.

Locked-Test Subset	n	R^2^
All compounds	1038	0.5831
Ki-only	652	0.6053
IC_50_-only	369	0.5097
Both endpoints	17	0.5060
No reactive alert	934	0.5717
At least one reactive alert	104	0.5674

**Table 9:** Summary of Y-randomization and applicability domain analyses.

Analysis	Parameter	Value
Y-randomization	Permutations	100
Y-randomization	Mean randomized R^2^	−0.1233
Y-randomization	SD randomized R^2^	0.0495
Y-randomization	Maximum randomized R^2^	−0.0033
Y-randomization	Empirical *p*-value	0.0099
Applicability domain	D*	0.5867
Applicability domain	Inside/outside compounds	962/76
Applicability domain	Coverage	92.68%
Applicability domain	Inside-domain R^2^/RMSE	0.6041/0.9268
Applicability domain	Outside-domain R^2^	−0.0043

Note: D* denotes the predefined applicability-domain distance threshold, calculated as the mean development nearest-neighbor distance plus three standard deviations.

## Data Availability

The data presented in this study are available in the ChEMBL database (European Molecular Biology Laboratory–European Bioinformatics Institute, EMBL-EBI) under the target identifier CHEMBL204. These data were derived from the publicly accessible ChEMBL database. The curated datasets and relevant analysis outputs supporting the findings of this study are provided in the Supplementary Materials.

## Supplementary Materials

The following supporting information can be downloaded at: https://www.mdpi.com/article/10.3390/ph19091411/s1, Figure S1: Endpoint composition after assay-aware curation; Figure S2: Agreement of paired Ki and IC_50_ summaries and the predefined exclusion boundary; Figure S3: Within-structure, within-endpoint replicate concordance; Figure S4: Development-fitted feature-generation audit; Figure S5: Locked scaffold split and test-to-development similarity; Figure S6: Endpoint and activity balance across scaffold partitions; Figure S7: Development scaffold-grouped out-of-fold model shootout; Figure S8: Global TreeSHAP importance for the dominant ExtraTreesMorgan component; Figure S9: Development-only Mordred permutation importance for the SVR component; Figure S10: Representative best, median, and worst random-test predictions; Table S1: Record-level curation flow; Table S2: Replicate and endpoint audit; Table S3: Feature filtering; Table S4: Partition balance; Table S5: Development scaffold-OOF ranking; Table S6: Hyperparameter optimization records; Table S7: Primary and sensitivity metrics.

## Abbreviations

The following abbreviations are used in this manuscript:

QSAR	Quantitative Structure–Activity Relationship
ChEMBL	Chemical Database of Bioactive Molecules (EMBL-EBI)
XAI	eXplainable Artificial Intelligence
Ki	Inhibition Constant
IC50	Half-Maximal Inhibitory Concentration
