# Supplementary material for: An Explainable Machine Learning-Based QSAR Framework for Predicting Thrombin Inhibitory Activity
Source: Pharmaceuticals (Basel). 2026 Sep 7;19(9):1411. doi: 10.3390/ph19091411 (PMC13610635; doi:10.3390/ph19091411)
Supplement: Supplementary file 1 [file pharmaceuticals-19-01411-s001.zip › pharmaceuticals-4529984-supplementary.pdf]

Supplementary methods and audit trail

This document summarizes the principal supplementary tables, figures, and audit records supporting the revision. The tables and figures report the curation flow, replicate and endpoint audits, predefined partition characteristics, feature-processing results, model-selection and validation outcomes, robustness analyses, and structural interpretations. The fixed project seed was 20260823. The locked scaffold-test responses were evaluated only after the final model candidate had been frozen.

Table S1. Record-level curation flow

Step	Records retained
Raw CHEMBL204 export	9327
Exact relation (=)	6849
Standard unit nM	6777
No validity warning	6435
Valid standardized structure	6434
Exact duplicate records removed	6414
Final unique pooled compounds	5189

Table S2. Replicate and endpoint audit

Category	n
Single-measurement structure–endpoint groups	4843
Highly consistent replicate groups (span ≤0.5)	451
Acceptable replicate groups (0.5<span≤1.0)	80
Conflicting replicate groups excluded (span>1.0)	96
Both-endpoint pairs	136
Conflicting cross-endpoint pairs excluded	49
Concordant both-endpoint structures retained	87

Table S3. Feature filtering

Representation	Stage	Features
Mordred	Calculated 2D	1613
Mordred	Missingness ≤20%	1450
Mordred	Nonzero variance	1312
Mordred	|r| ≤0.95 retained	615
Morgan radius 2, 2048 bits	Nonconstant development bits	2039

Table S4. Partition balance

Partition	Compounds	Scaffolds	Mean pActivity	SD	Reactive alerts	Ki-only	IC_{50}-only	Both
Development	4151	1180	6.5613	1.4723	416	2607	1474	70
Locked test	1038	295	6.5598	1.4697	104	652	369	17

Table S5. Development scaffold-OOF ranking

Model	View	R^2	RMSE	MAE	CCC
ConsensusTop5	Ensemble	0.5983	0.9331	0.7230	0.7268
ConsensusAll	Ensemble	0.5975	0.9340	0.7238	0.7264
ConsensusTop3	Ensemble	0.5834	0.9502	0.7332	0.7205
ExtraTreesMorgan	Morgan	0.5714	0.9637	0.7330	0.7245
XGBoostCombined	Combined	0.5447	0.9933	0.7767	0.6891
LightGBMCombined	Combined	0.5369	1.0018	0.7834	0.6789
NuSVRMorgan	Morgan	0.5204	1.0195	0.7946	0.6810

Table S6. Hyperparameter optimization records

All configurations were selected using development-only folds. Numerical values are rounded for presentation.

Table S6A. Scaffold-grouped development selection.

Model	Selected configuration
Ridge	alpha = 894.167
ElasticNet	alpha = 0.0240; l1_ratio = 0.2799
PLS	n_components = 5
SVR	C = 27.8287; epsilon = 0.2188; gamma = 0.002662
RandomForest	n_estimators = 350; max_depth = 16; min_samples_split = 5; min_samples_leaf = 2; max_features = 0.5026
ExtraTrees	n_estimators = 350; max_depth = 36; min_samples_split = 10; min_samples_leaf = 2; max_features = 0.4926; bootstrap = False
HistGradientBoosting	learning_rate = 0.06264; max_iter = 350; max_leaf_nodes = 95; max_depth = 6; min_samples_leaf = 36; l2_regularization = 0.01006
XGBoostCombined	n_estimators = 650; learning_rate = 0.05897; max_depth = 5; min_child_weight = 1.1880; subsample = 0.9147; colsample_bytree = 0.4480; reg_alpha = 8.21e-6; reg_lambda = 0.2484
LightGBMCombined	n_estimators = 550; learning_rate = 0.02916; num_leaves = 43; max_depth = 16; min_child_samples = 34; subsample = 0.6780; colsample_bytree = 0.7295; reg_alpha = 0.02441; reg_lambda = 0.003058
CatBoostMordred	iterations = 750; learning_rate = 0.09187; depth = 6; l2_leaf_reg = 2.0906; random_strength = 0.02217
NuSVRMorgan	nu = 0.6805; C = 17.0581; gamma = 0.0003440
ExtraTreesMorgan	n_estimators = 200; max_depth = 40; min_samples_split = 5; min_samples_leaf = 1; max_features = 0.2653
ConsensusTop5	weights: ExtraTreesMorgan = 0.4415; XGBoostCombined = 0.0860; LightGBMCombined = 0; NuSVRMorgan = 0.2191; SVR = 0.2534

Table S6B. Random-split development selection.

Model	Selected configuration
ExtraTreesMorgan	n_estimators = 400; max_depth = None; min_samples_split = 4; min_samples_leaf = 1; max_features = 0.2635
XGBoostCombined	n_estimators = 650; learning_rate = 0.02877; max_depth = 7; min_child_weight = 1.2264; subsample = 0.8498; colsample_bytree = 0.5439; reg_alpha = 5.39e-5; reg_lambda = 0.001179
LightGBMCombined	n_estimators = 750; learning_rate = 0.03523; num_leaves = 24; max_depth = -1; min_child_samples = 33; subsample = 0.8848; colsample_bytree = 0.6908; reg_alpha = 0.09967; reg_lambda = 5.6524
SVRMordred	C = 28.8919; epsilon = 0.2557; gamma = 0.001934
NuSVRMorgan	nu = 0.5793; C = 8.4095; gamma = 3.6246e-5
ConsensusAll	weights: ExtraTreesMorgan = 0.6258; XGBoostCombined = 0.0145; LightGBMCombined = 0; SVRMordred = 0.2876; NuSVRMorgan = 0.0721

Table S7. Primary and sensitivity metrics

Analysis	n	R^2	RMSE	MAE
Locked scaffold test	1038	0.5831	0.9484	0.7290
Locked Ki-only	652	0.6053	0.9421	0.7146
Locked IC_{50}-only	369	0.5097	0.9596	0.7542
Locked both-endpoint	17	0.5060	0.9466	0.7317
Locked no-alert	934	0.5717	0.9495	0.7300
Locked reactive-alert	104	0.5674	0.9385	0.7196
AD inside	962	0.6041	0.9268	0.7096
AD outside	76	−0.0043	1.1890	0.9743
Random held-out	1038	0.7480	0.7360	0.5307

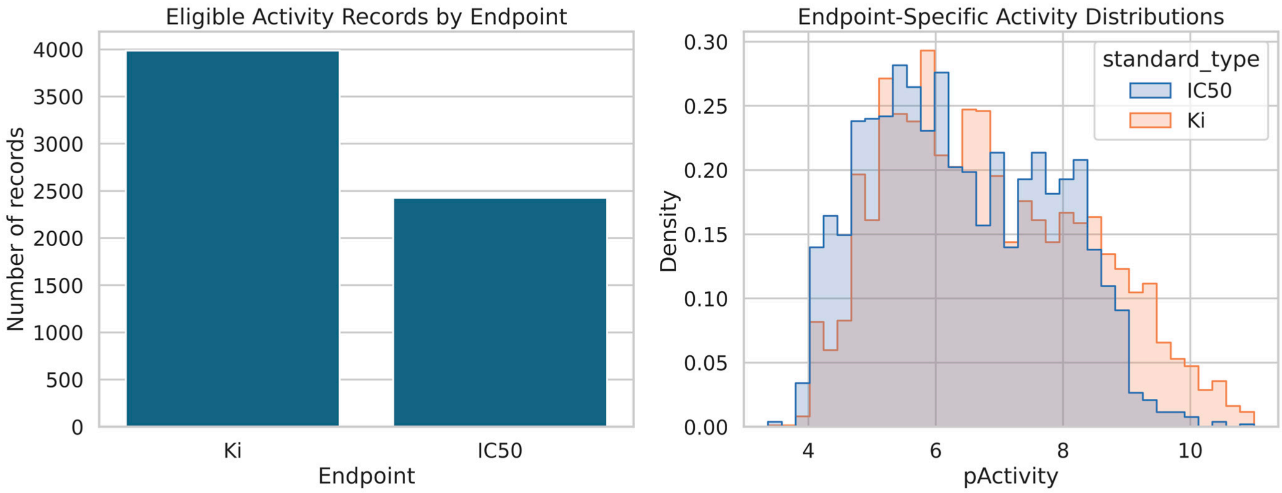

Figure S1. Endpoint composition after assay-aware curation.

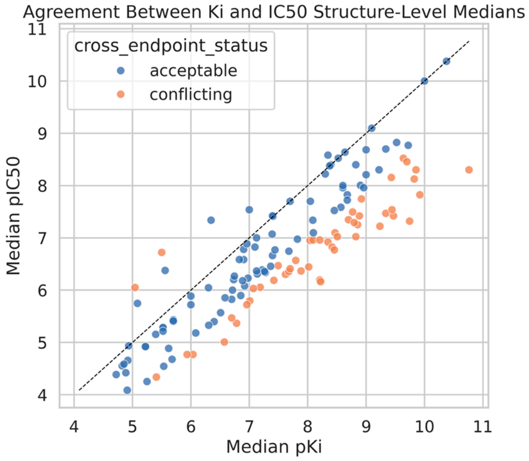

Figure S2. Agreement of paired Ki and IC₅₀ summaries and the predefined exclusion boundary.

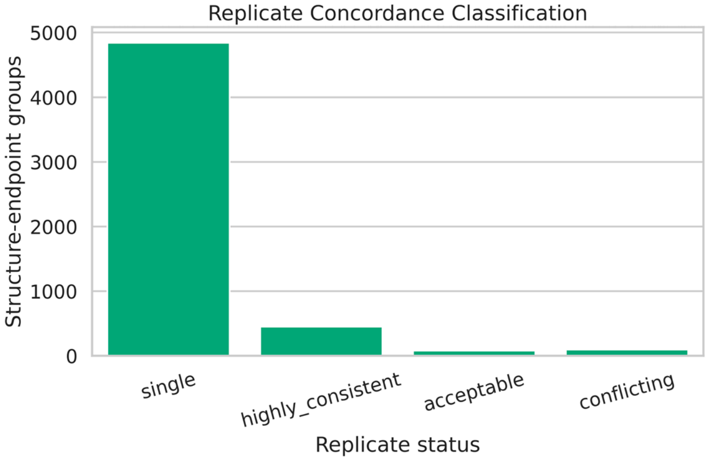

Figure S3. Within-structure, within-endpoint replicate concordance.

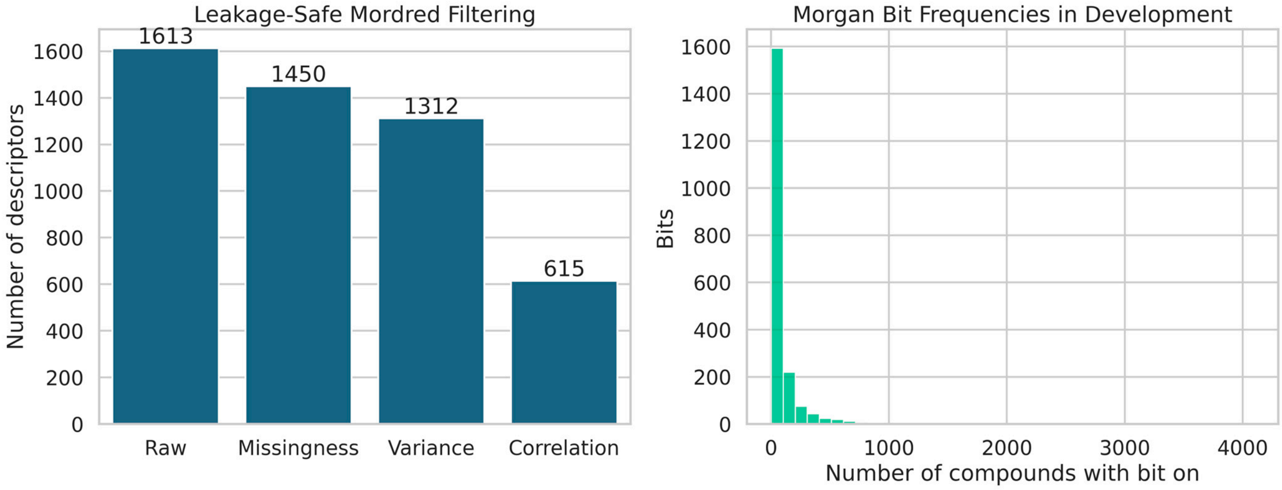

Figure S4. Development-fitted feature-generation audit.

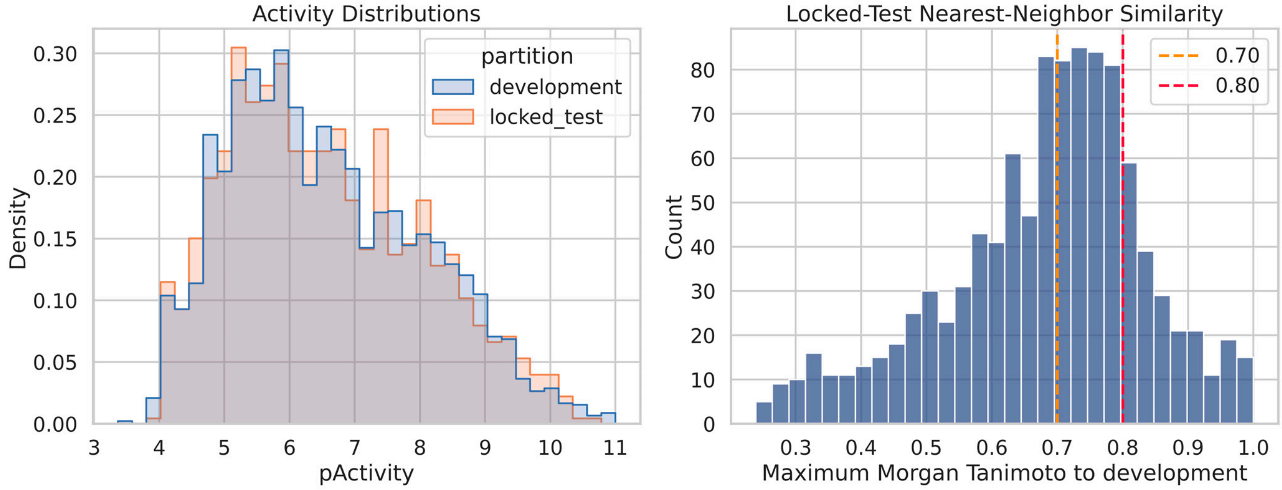

Figure S5. Locked scaffold split and test-to-development similarity.

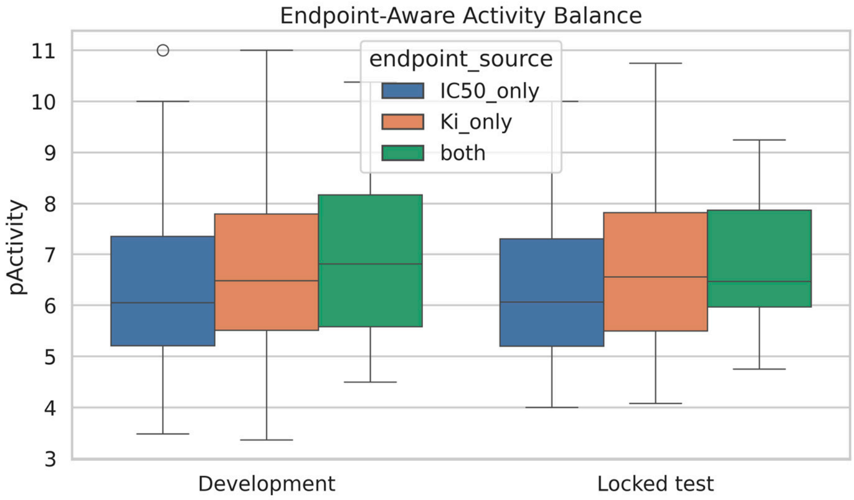

Figure S6. Endpoint and activity balance across scaffold partitions.

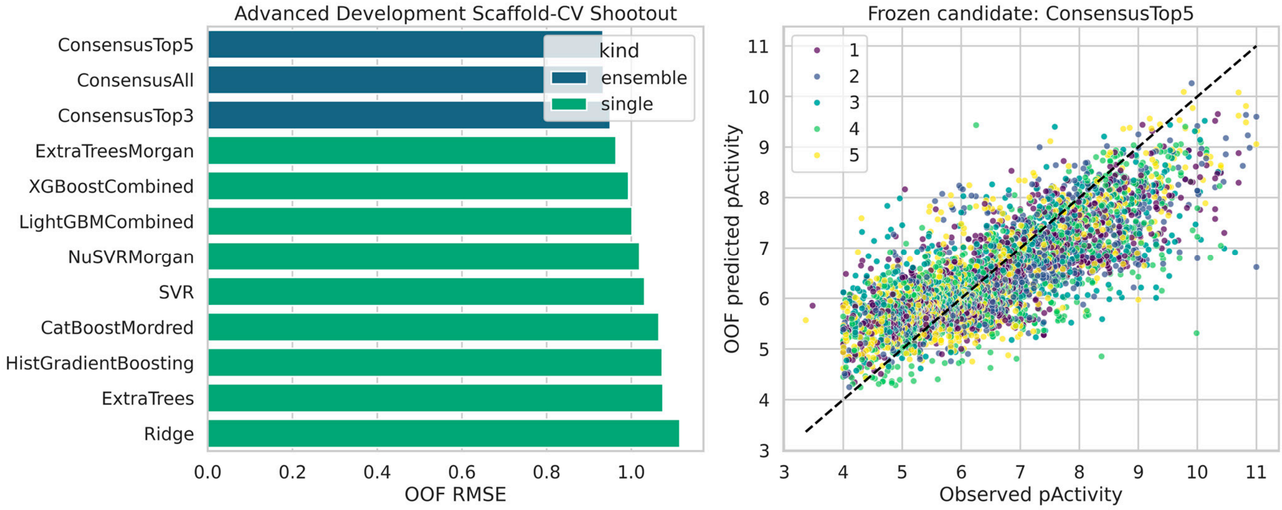

Figure S7. Development scaffold-grouped out-of-fold model shootout.

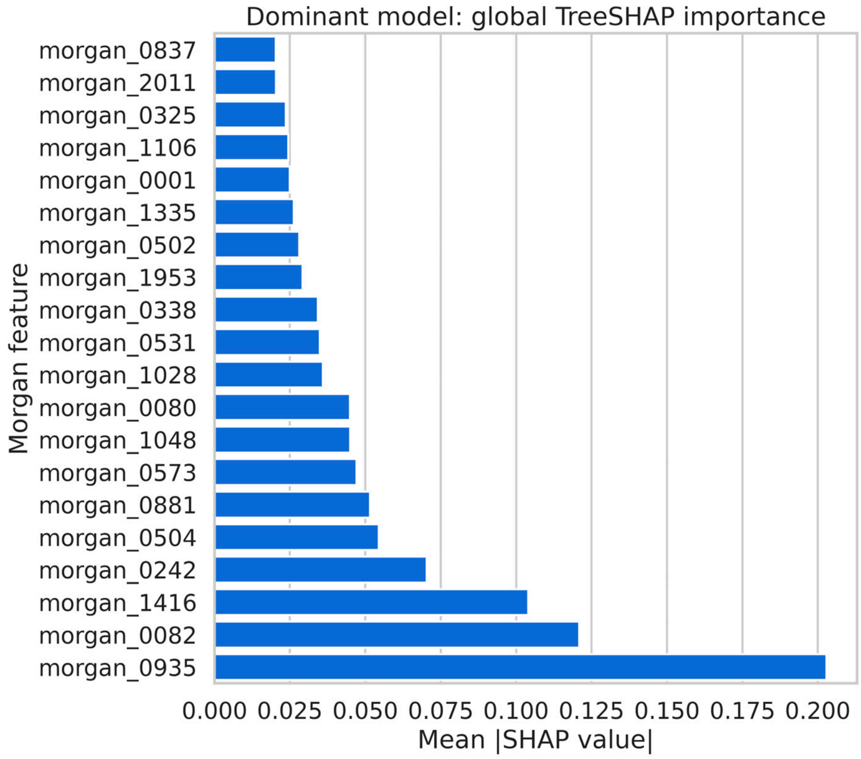

Figure S8. Global TreeSHAP importance for the dominant ExtraTreesMorgan component.

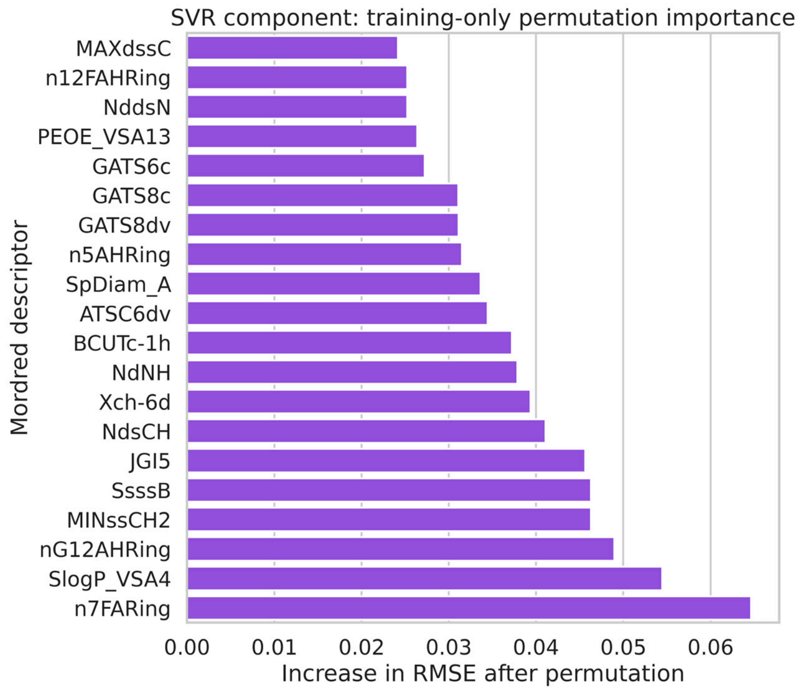

Figure S9. Development-only Mordred permutation importance for the SVR component.

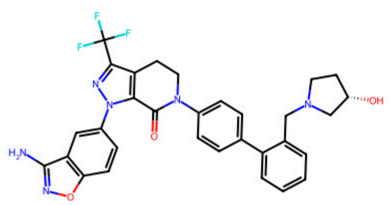

THR_02967
Obs 7.23 | Pred 7.23

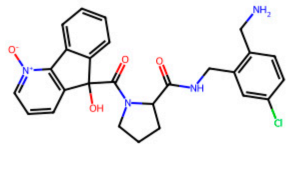

THR_02305
Obs 8.58 | Pred 8.58

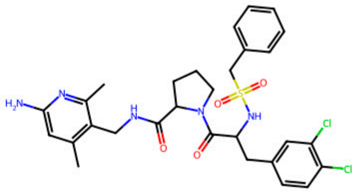

THR_02587
Obs 7.96 | Pred 7.96

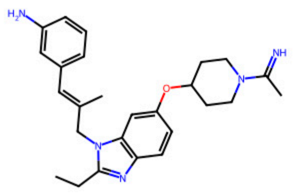

THR_03540
Obs 6.42 | Pred 6.43

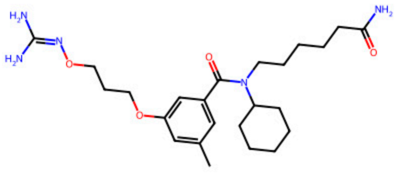

THR_02526
Obs 8.10 | Pred 7.72

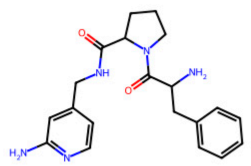

THR_03537
Obs 6.42 | Pred 6.80

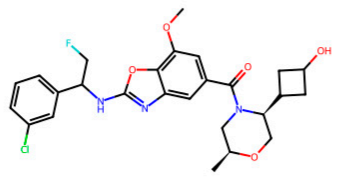

THR_00009
Obs 9.43 | Pred 9.05

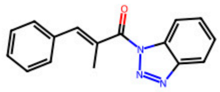

THR_00905
Obs 6.07 | Pred 5.69

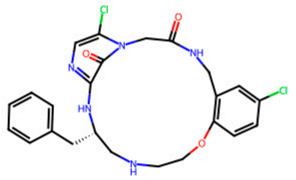

THR_03649
Obs 6.25 | Pred 9.98

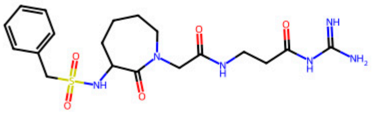

THR_01800
Obs 4.14 | Pred 7.75

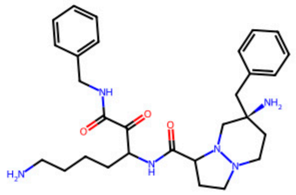

THR_04128
Obs 5.70 | Pred 8.75

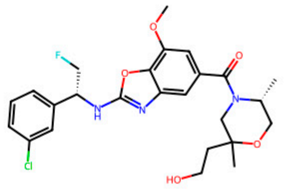

THR_00001
Obs 11.00 | Pred 8.08

Figure S10. Representative best, median, and worst random-test predictions.
